# Effects of Nasal Corticosteroids on Boosts of Systemic Allergen-Specific IgE Production Induced by Nasal Allergen Exposure

**DOI:** 10.1371/journal.pone.0114991

**Published:** 2015-02-23

**Authors:** Cornelia Egger, Christian Lupinek, Robin Ristl, Patrick Lemell, Friedrich Horak, Petra Zieglmayer, Susanne Spitzauer, Rudolf Valenta, Verena Niederberger

**Affiliations:** 1 Division of Immunopathology, Department of Pathophysiology and Allergy Research, Center for Pathophysiology, Infectiology and Immunology, Medical University of Vienna, Vienna, Austria; 2 Section for Medical Statistics, Center for Medical Statistics, Informatics, and Intelligent Systems, Medical University of Vienna, Vienna, Austria; 3 Department Vienna Challenge Chamber, Allergy Centre Vienna West, Vienna, Austria; 4 Department of Medical and Chemical Laboratory Diagnostics, Medical University of Vienna, Vienna, Austria; 5 Department of Otolaryngology, Vienna General Hospital, Medical University of Vienna, Vienna, Austria; Beijing Institiute of Otolaryngology, CHINA

## Abstract

**Background:**

Allergen exposure via the respiratory tract and in particular via the nasal mucosa boosts systemic allergen-specific IgE production. Intranasal corticosteroids (INCS) represent a first line treatment of allergic rhinitis but their effects on this boost of allergen-specific IgE production are unclear.

**Aim:**

Here we aimed to determine in a double-blind, placebo-controlled study whether therapeutic doses of an INCS preparation, i.e., nasal fluticasone propionate, have effects on boosts of allergen-specific IgE following nasal allergen exposure.

**Methods:**

Subjects (n = 48) suffering from grass and birch pollen allergy were treated with daily fluticasone propionate or placebo nasal spray for four weeks. After two weeks of treatment, subjects underwent nasal provocation with either birch pollen allergen Bet v 1 or grass pollen allergen Phl p 5. Bet v 1 and Phl p 5-specific IgE, IgG_1–4_, IgM and IgA levels were measured in serum samples obtained at the time of provocation and one, two, four, six and eight weeks thereafter.

**Results:**

Nasal allergen provocation induced a median increase to 141.1% of serum IgE levels to allergens used for provocation but not to control allergens 4 weeks after provocation. There were no significant differences regarding the boosts of allergen-specific IgE between INCS- and placebo-treated subjects.

**Conclusion:**

In conclusion, the application of fluticasone propionate had no significant effects on the boosts of systemic allergen-specific IgE production following nasal allergen exposure.

**Trial Registration:**

http://clinicaltrials.gov/ NCT00755066

## Introduction

Immunoglobulin E (IgE) plays a central role in the pathogenesis of allergy and asthma. Allergen-induced cross-linking of IgE bound to the surface of mast cells and basophils via FcεRI leads to the degranulation of these cells and the release of inflammatory mediators, proteases and pro-inflammatory cytokines [1]. IgE also enhances allergen uptake and presentation to T cells by antigen presenting cells (dendritic cells, monocytes and B cells) via binding to FcεRI and the low affinity IgE receptor FcεRII (CD23) [2,3].

In addition, IgE prolongs the survival of mast cells and up-regulates the expression of its receptors (FcεRI, CD23) [4]. Furthermore, it has been demonstrated that mast cell and basophil sensitivity correlates with the levels of allergen-specific IgE antibodies [5, 6].

Several clinical studies have demonstrated that recurrent allergen contact increases the levels of allergen-specific IgE antibodies and the clinical sensitivity towards the corresponding allergens [7–12], whereas prolonged lack of allergen contact will decrease allergen-specific IgE and eventually lead to clinical unresponsiveness [13]. In this context it was shown that antigen/allergen stimulation particularly via the nasal mucosa is followed by an increase of allergen-specific IgE levels [11, 14–15].

For allergen-specific immunotherapy (SIT) it was demonstrated that the induction of allergen-specific IgG was associated with a reduction of the boosts of allergen-specific IgE production after allergen exposure, suggesting that SIT has a suppressive effect on allergen-specific IgE production [16–19].

Intranasal corticosteroids (INCS) represent a first line anti-inflammatory drug used for the treatment of allergic rhinitis but their underlying effects on the allergic immune response are not entirely clear. While the anti-inflammatory properties of corticosteroids are well studied, less is known about their impact on allergen-specific IgE levels. *In vitro* studies using cultured peripheral blood mononuclear cells (PBMC) have demonstrated that corticosteroids enhance interleukin (IL)-4-induced rises of IgE levels [20–23]. Similar observations were made in allergic patients, who exhibited a polyclonal rise of IgE antibodies in their sera after systemic treatment with prednisolone [24]. On the other hand, corticosteroids have been shown to selectively reduce rises of nasal IL-4, IL-5 and IL-13-producing cells following *in vivo* allergen exposure [25], thereby possibly being capable of down-regulating IgE production. A few studies which investigated the effects of topical corticosteroids on IgE production showed either no or a dampening effect [26–28].

In the present double-blind placebo-controlled study we used purified recombinant allergens for controlled nasal provocation in allergic subjects to investigate whether treatment with a frequently used topical corticosteroid, i.e., nasal fluticasone propionate, impacts on systemic allergen-specific IgE levels following nasal allergen exposure.

## Methods

The protocol for this trial and supporting CONSORT checklist are available as supporting information; see S1 CONSORT Checklist and S1 Protocol. The study was approved by the ethical committee of the Medical University of Vienna, the etical committee of the "Österreichischen Arbeitsgemeinschaft für klinische Pharmakologie und Therapie" and the ethical committee of the private hospital "Institut für Hypertoniker" (1090 Vienna, Kinderspitalgasse 10/15). All study participants gave written informed consent. The study has been registered at http://clinicaltrials.gov/ under the trial number: NCT00755066. Inclusion of patients was started before registration at clinicaltrials.gov because the importance to do so was unknown to the investigators at the time. The study was however registered at the EudraCT website before inclusion of patients was initiated (Eudract-number: 2005-004274-24). Participants were included in the study between November 2005 and February 2006. The authors confirm that all ongoing and related trials for this drug/intervention are registered

### Recombinant allergens

Recombinant pollen allergens (rPhl p 1, rPhl p 5, rBet v 1), were obtained from BIOMAY (Vienna, Austria). rPhl p 1 [29] and rPhl p 5 [30] represent two major timothy grass pollen allergens and rBet v 1 [31] is the major birch pollen allergen. The allergens were selected because they are recognized by the vast majority of patients sensitized to the corresponding allergen sources and are immunologically distinct from each other.

### Study design

**Fig 1:**
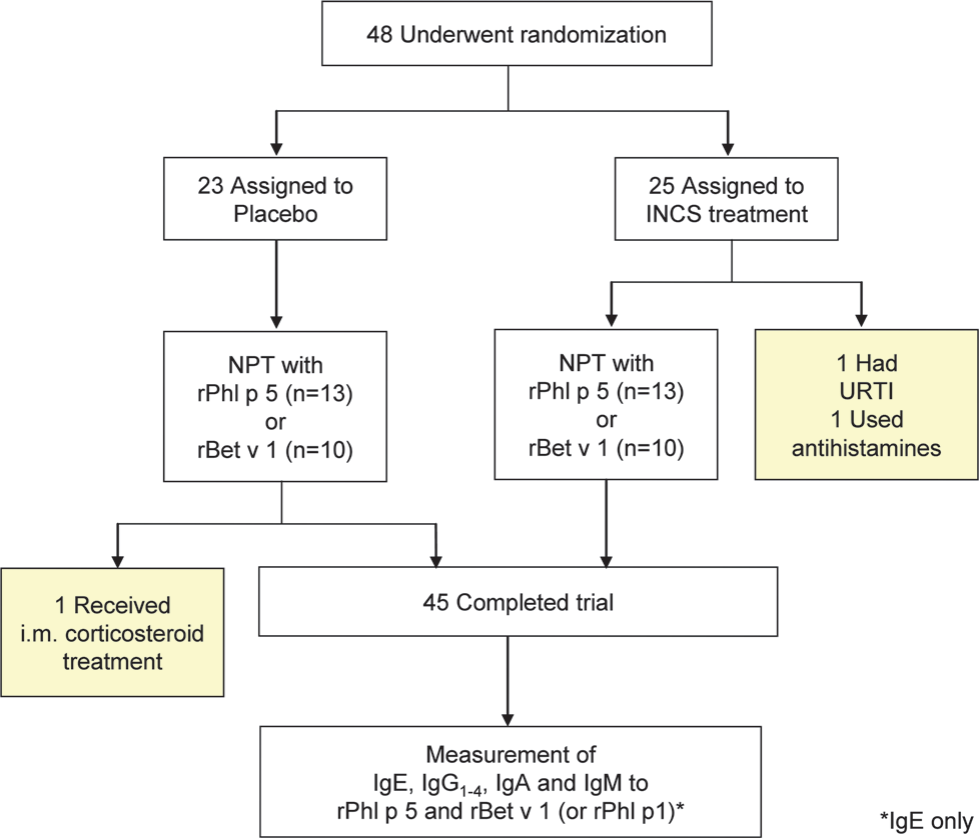
Distribution of study subjects. Of the 48 volunteers who were randomized, 45 completed the trial. Drop-outs are marked in yellow. INCS: intranasal corticosteroid; NPT: nasal provocation test; URTI: upper respiratory tract infection; i.m.: intra-muscular

**Fig 2:**
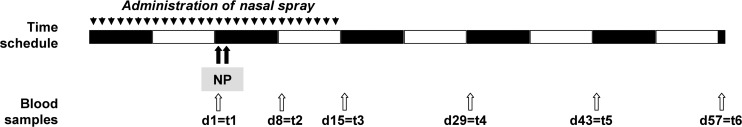
Study design. Patients administered nasal spray containing either nasal fluticasone propionate or placebo (black arrows) for 2 weeks before and after nasal allergen provocation (NP) performed on two consecutive days. Blood samples for measurement of allergen-specific antibodies (white arrows) were obtained at the time of the first nasal provocation (day 1 = t1) and on days 8 (t2), 15 (t3), 29 (t4), 43 (t5) and 57 (t6).

Forty eight grass and/or birch pollen allergic subjects (Fig. 1, Table 1) who were sensitized to rPhl p 5 and/or rBet v 1 were enrolled in a randomized, double-blind, placebo-controlled study. Volunteers were recruited using posters to advertise the study at the Medical University of Vienna. The study design is depicted graphically in Fig. 2. Study participants were randomized to receive four weeks of either fluticasone propionate (100μg daily into each nostril, corresponding to the recommended daily treatment dose of 200μg) or placebo nasal spray. After two weeks of treatment, subjects underwent nasal provocation with either rPhl p 5 or rBet v 1 on two consecutive days. A pre-treatment phase of 2 weeks before allergen exposure was chosen in order to comply with the ARIA guidelines (www.whiair.com) which suggest that the maximum efficacy of corticosteroids requires up to two weeks of treatment. Serum IgE levels to the allergen used for nasal provocation (rPhl p 5 or rBet v 1) and to a control allergen (rBet v 1 or rPhl p 5 or rPhl p 1, respectively) were the primary outcome measure and were determined in blood samples obtained on the day of nasal provocation and 1, 2, 4, 6 and 8 weeks thereafter. The whole study was performed in winter outside the birch and grass pollen season to exclude effects due to natural seasonal allergen contact.

**Table 1:** Demographic and clinical data of study participants.

	Fluticasone propionate	Placebo
Characteristic	(N = 23)	(N = 22)
**Male sex**—no (%)	15 (65)	10 (45)
**Age—yr**		
Mean	26.1±4.5	25.2±4.1
Range	20–40	19–37
**No. of years with seasonal rhinitis**	14.3±4.7	11.7±6.1
**Specific IgE (kUA/l)**		
***Allergen used for nasal provocation***		
Median	11.6	11.0
IQR	20.7	24.4
***Control allergen***		
Median	7.1	6.7
IQR	13.3	17.1
**Skin prick test—wheal area (mm2)**		
***Allergen used for nasal provocation***	40.3	47.2
***Control allergen***	37.6	47.1
**Symptom score during previous pollen season**		
***Allergen used for nasal provocation***	9.5±3.4	8.7±4.2
***Control allergen***	8.2±3.7	7.0±4.0
**Total IgE (kU/l)**		
Median	197	196
IQR	235.3	233.0
**Sensitivity to other allergens—no. (%)**		
Dust mites	10 (43)	17 (77)
Cat dander	8 (35)	11 (50)
Mold	2 (9)	3 (14)
**Coexisting conditions—no. (%)**		
Asthma	4 (17)	5 (23)
Conjunctivitis	23 (100)	21 (95)

*Plus-minus values are means +/- SD. Wheal areas were determined by computer-aided planimetry. IQR = Interquartile range.

### Study subjects and study medication

All 48 volunteers who participated in the study were grass and/or birch pollen allergic as assessed by case history, standard skin prick testing with inhalant allergens and ImmunoCAP measurements (Phadia, Uppsala, Sweden). All but one of the subjects was sensitized to at least two of the three allergen molecules. Subjects’ demographic and clinical data including standard test results and specific IgE levels to recombinant allergens are shown in Table 1. Twenty-five subjects were randomized to be treated with fluticasone propionate nasal spray and 23 subjects were assigned to placebo (Fig. 1). Randomized study medication was provided in blocks of four by GlaxoSmithKline and decoding information was kept in a sealed envelope which was opened for de-blinding only in case of a systemic adverse reaction and after database closure was completed and all data were deposited with an independent statistician. The compliance of the study participants was ensured by weighing all treatment bottles after two and four weeks of treatment. All subjects had stable lung function and did not receive antihistamines or corticosteroids other than the study drug during the study. Corticosteroids were not allowed within three months and antihistamines were not allowed two weeks prior to the study. Subjects suffering from perennial allergic rhinitis and signs of airway infection during the treatment period were excluded from the study.

### Nasal provocation

At the beginning of the study all study participants were asked about the severity of their nasal symptoms during the previous birch and grass pollen season. Symptoms (i.e., nasal itching, sneezing, rhinorrhea, nasal blockage) were graded as follows: 0 points: not present; 1 point: present but no discomfort; 2 points: present, some discomfort, not interfering with daily life activities; 3 points: bothersome and interfering with daily life activities or disturbing sleep [32]. The use of medication to control symptoms received 2 additional points.

Based on this score the allergen from the source which had caused more severe symptoms was chosen for nasal provocation. Twenty-six subjects were assigned to nasal provocation with rPhl p 5 and 20 subjects to nasal provocation with rBet v 1 (Fig. 1). Nasal provocation with recombinant allergens was performed as previously described [11, 33]. Subjects underwent nasal application of the same allergen on two consecutive days after a two week period of treatment with either fluticasone propionate or placebo (Fig. 2). Purified recombinant allergens were diluted to 20μg/ml in sterile 0.9% sodium chloride solution and administered using a metered-dose nasal pump spray delivering 40 μl of solution per spraying action. On each day of nasal provocation, two sprays of the solution were given into one nostril, resulting in a dose of 1.6μg of allergen per study day and in a cumulative dose of 3.2 μg of recombinant allergen per subject. The dose used for nasal provocation was chosen because it has been used in previous studies [11] and because it is in the same range as the daily dose of allergen which a patient would be exposed to during high pollen season [34]. In fact, it has been shown that during the peak pollen season, a grass pollen allergic patient inhales approximately 5000 pollen grains per day, corresponding to 0.6μg of Phl p 5 allergen [34]. As Phl p 5 represents only one of 11 allergens contained in grass pollen [35], the amount of allergen used by us (i.e., 1.6μg of one recombinant allergen per study day) is in a dose range occurring during natural exposure.

Measurement of changes in nasal flow and nasal symptoms were recorded merely in order to ensure that nasal provocation was successful in all subjects. Therefore, these data were only a secondary endpoint of this study. Nasal flow was determined by active anterior rhinomanometry at 150 Pa measured before administration of test substances, 10 minutes after application of 0.9% sodium chloride, and 10 and 20 minutes after administration of test substances. Flow reduction was calculated by subtracting nasal flow after administration of test substances from nasal flow after administration of 0.9% sodium chloride solution. Subjective nasal symptoms after provocation were determined by adding scores (maximum: 12 scores) for the symptoms nasal itching, nasal congestion and nasal discharge using a 4-point scale (0: no symptoms, 1: mild symptoms, 2: moderate symptoms, 3: severe symptoms) and by counting the number of sneezes (0: no sneezes, 1: ≤5 sneezes, 2: 6–10 sneezes, 3: >10 sneezes) [36, 37]. In addition, subjects recorded how long it took for their nasal symptoms to subside after nasal provocation.

### Blood samples, measurement of antibodies

For the preparation of serum, blood samples were taken by puncture of the antecubital vein on the day of nasal provocation as well as 1, 2, 4, 6 and 8 weeks thereafter (Fig. 2). Serum samples were stored at -20°C until use and analyzed for IgE, IgA, IgM and IgG_1–4_ antibodies to the allergen used for nasal provocation and to an unrelated control. For subjects who had received rPhl p 5 by nasal provocation, rBet v 1 or rPhl p 1 served as control allergen, whereas for subjects who had received rBet v 1 for nasal provocation, rPhl p 5 or rPhl p 1 were used as control allergens (control allergen: Phl p 1: n = 17; Bet v 1: n = 8; Phl p 5: n = 19; no control allergen: n = 1).

Total and allergen-specific IgE levels were determined by ImmunoCAP measurements (Phadia, Uppsala, Sweden). All serum samples collected during the study were measured at the end of the study in a blinded manner. Allergen-specific IgA, IgM and IgG_1–4_ levels were measured by ELISA as described [33].

The six serum samples obtained from each subject during the course of the study were analyzed for each isotype and subclass against each of the recombinant allergens on one plate to assess accurately the time course of antibody responses. Each serum sample was tested in duplicate and mean values of the optical densities (OD) were calculated. Differences between duplicate wells were consistently <10%. Reference sera for each isotype and subclass were included on each plate to avoid plate-to-plate variations.

### Statistical analysis

Statistical analysis was performed using IBM SPSS Statistics, Version 20.0 (IBM Corp. Armonk, NY) and SAS 9.4 (SAS Institute Inc., Cary, NC, USA). Sample size calculations were based on the following assumptions: It was expected that during the eight weeks period rPhl p 5/rBet v 1-specific serum IgE antibody levels would increase after challenge to a level of at least one standard deviation above baseline [11]. Our hypothesis was that this increase would be prevented by INCS by a true effect of at least 1 standard deviation at one time point. Assuming a moderate correlation of 0.4 between repeated measurements within a patient, a sample size of 19 per group (placebo, fluticasone) was calculated to result in a power of 80% to detect the assumed difference between groups using an F-Test from a repeated measures design at a significance level of 0.05. A higher number of patients was included to account for possible drop-outs.

The changes in Phl p 5- and Bet v 1-specific IgE-levels between t1 and t2, t3, t4, t5 and t6, respectively, were assessed by linear mixed effect models. The data were log-transformed before the analyses, due to the right skewed distribution of the raw data. The differences between the IgE values at time-points t2 to t6, respectively, and the baseline value at t1 were calculated for the log-transformed data. A linear mixed model was fit to explain the differences in log-IgE values by the categorical variable time-point, treatment group (placebo vs. fluticasone) and the interaction between time-point and treatment. A random intercept for the patient was included to account for the within-patient correlation of repeated observations.

From this model, the null hypothesis of no effect of time and the null hypothesis of no treatment effect at any time point were tested by F-tests.

The effect of treatment was estimated at each time-point as the mean log-IgE difference in the fluticasone group minus the mean log-IgE difference in the placebo group. A corresponding 95% confidence interval was calculated. In addition, the average effect of treatment on the log IgE difference over all five time-points was estimated.

Following back-transformation, the estimated effects can be interpreted as median of the ratio of relative changes from t1.

The analysis was performed for Phl p 5-specific IgE levels, Bet v 1-specific IgE and combined Phl p 5- and Bet v 1-specific IgE levels.

Including the baseline (t1) value of log-IgE in the regression model was considered, to account for a possible influence of the initial amount of IgE on the change. However, for both, Phl p 5 and Bet v 1, and the combination of both allergens the effect of baseline IgE was not significant and small in magnitude and it was not included in the final models.

The calculations were done using PROC Mixed in SAS 9.4. The method due to Kenward and Roger was used to calculate degrees of freedom and standard errors.

## Results

### Study participants

Forty-eight birch and grass pollen allergic subjects participated in the study and were randomized to the two treatment groups (Table 1; Fig. 1). Two subjects from the actively treated group were excluded from the study at the time of nasal provocation, one because he had used oral antihistamines, and the other because he had a viral upper respiratory tract infection. Another subject from the placebo group was excluded after nasal provocation because he had received intramuscular corticosteroids for a medical problem that was unrelated to the study (Fig. 1).

Demographic characteristics of placebo- and INCS-treated subjects are displayed in Table 1. The two groups were comparable regarding demographic parameters, allergic history, symptoms as well as total and specific IgE levels to the allergens used for nasal provocation and the control allergens.

### Allergen-specific systemic IgE levels are boosted by nasal allergen exposure

After two weeks of treatment with 200μg fluticasone propionate or placebo nasal spray, 26 subjects were exposed to Phl p 5 and 20 subjects to Bet v 1 by nasal provocation (Fig. 2). Symptoms and flow reduction after nasal provocation were recorded to ensure that the provocation procedure was performed successfully in all patients but these results were not a primary outcome of this study. Nasal provocation was followed by an increase in the symptom scores in all 43 patients. There was however no relevant difference between those subjects who had been pre-treated with steroid and placebo nasal spray regarding their nasal symptoms after the first nasal provocation (median placebo: 7 scores, median steroid: 6 scores, n.s.) and second nasal provocation (median placebo: 6 scores, median steroid: 6 scores, n.s.). Also, we observed a decrease in nasal flow in 21 of the 22 subjects in the placebo group (median decrease: day 1: 223 ml/s, p<0.001, day 2: 217 ml/s, p<0.001) and in 19 of the 21 subjects in the steroid treated group (median decrease: day 1: 185 ml/s, p<0.001, day 2: 167ml/s, p<0.001). There was a trend towards lower flow reduction in the INCS group which was however not statistically significant. Subjects recovered significantly more quickly from their nasal symptoms after nasal provocation if they had been pre-treated with nasal steroids (median time to recovery: placebo: 165 minutes, steroids: 60 minutes, p-value: 0.019).

**Fig 3:**
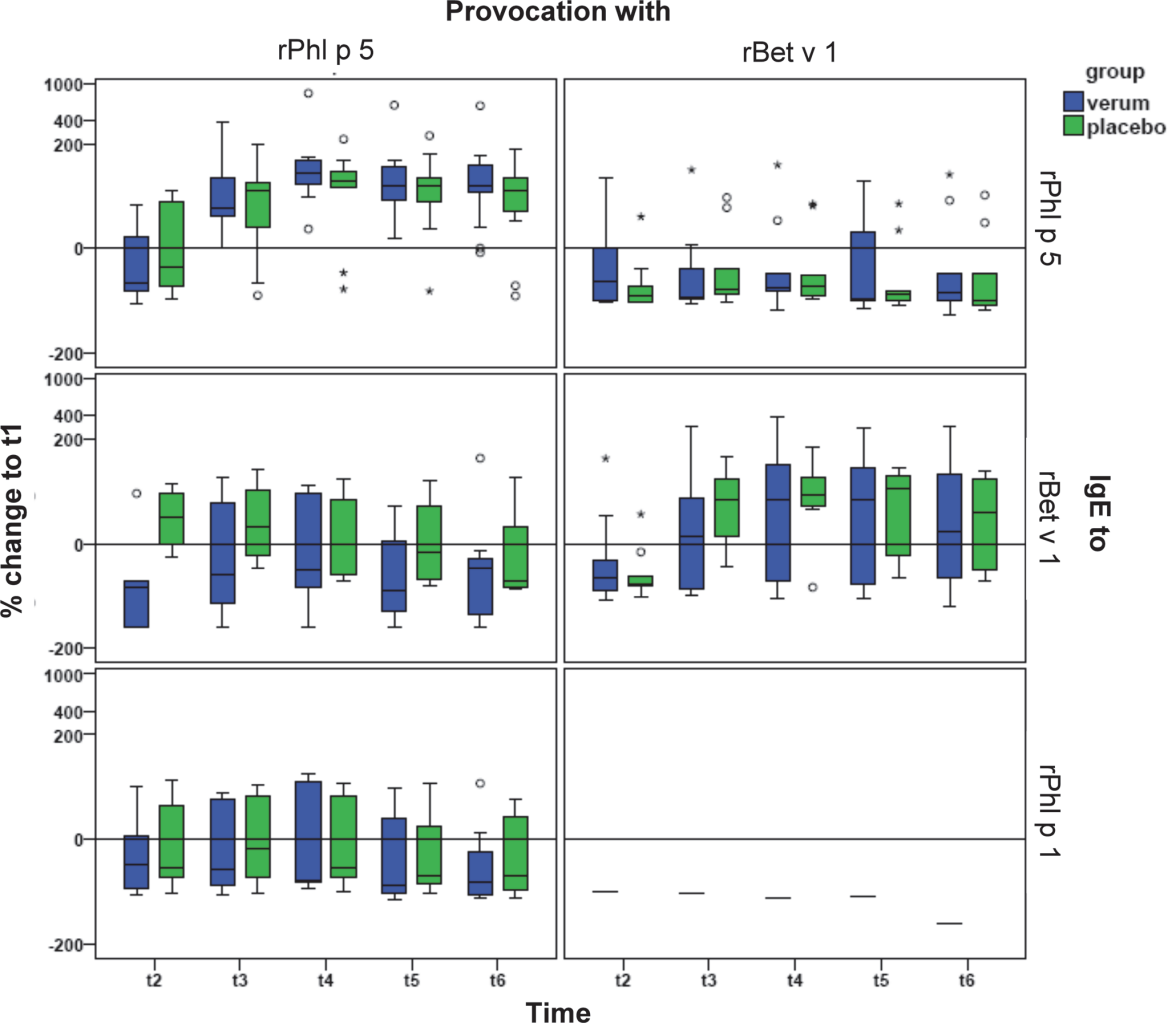
Development of allergen-specific IgE levels. Relative changes of IgE-levels (y-axes) to Phl p 5, Bet v 1 and Phl p 1 (top to bottom: right labels of each chart) compared to t1 (day 1) are shown for all visits (t2: day 8; t3: day 15; t4: day 29; t5: day 43; t6: day 57, x-axes). Results for the steroid-treated group are shown in blue, the placebo-group in green. Results from participants challenged with Phl p 5 are depicted in the left column, those for the Bet v 1-challenged group on the right side. Outliers that lie between 1.5 and 3 times the interquartile range below the first or above the third quartile are shown as open circles (“o”), those that lie beyond 3 times the interquartile range are depicted by asterisks (“*”).There were no significant differences between the fluticasone and placebo groups at any time point.

Systemic allergen-specific IgE-levels were the primary endpoint of this study. Two, 4, 6 and 8 weeks after nasal provocation we observed a substantial and statistically significant (p < 0.001) rise of allergen-specific IgE levels to the allergen used for nasal provocation but not to the control allergen (average of the median increases for Phl p 5 and Bet v 1 at t4: 141.1%, Fig. 3). The maximum rise of specific IgE was measured four weeks after provocation. This rise was followed by a plateau/slight decline of specific serum IgE levels until week 8 (Fig. 3). No relevant changes of IgE levels to the control allergens were observed (Fig. 3). There was no statistically significant difference between the placebo- and nasal steroid-treated group regarding the development of allergen-specific IgE (Fig. 3, Table 2). In more detail, the ratio of IgE increase in the fluticasone versus placebo treated patients was almost 1 at all time-points, indicating no difference, in both the group exposed to nasal Phl p 5, Bet v 1, and a combination of the two groups (Table 2, column: “Ratio of relative changes from t1”). A calculation of confidence intervals revealed that the risk of overlooking a beneficial average blunting effect of fluticasone on IgE increases of more than 14% was less than 2.5% (Table 2). Furthermore, no relevant correlations were found between the baseline IgE-levels at t1 and the magnitudes of the relative changes in IgE-levels in the subsequent observation period (data not shown).

**Table 2:** Effect of fluticasone versus placebo on the median relative change in allergen-specific IgE levels.

Allergen	Time-point	Ratio of relative changes from t1	95% CI low	95% CI up
Phl p 5	t2	0.899	0.654	1.236
	t3	1.064	0.775	1.459
	t4	1.220	0.890	1.674
	t5	1.091	0.796	1.497
	t6	1.189	0.865	1.634
	average	1.086	0.822	1.436
Bet v 1	t2	1.039	0.710	1.521
	t3	0.948	0.649	1.385
	t4	1.040	0.712	1.518
	t5	1.080	0.739	1.577
	t6	1.050	0.719	1.533
	average	1.030	0.727	1.460
Combined	t2	0.964	0.758	1.227
	t3	1.013	0.798	1.286
	t4	1.142	0.899	1.449
	t5	1.090	0.858	1.384
	t6	1.127	0.887	1.432
	average	1.065	0.861	1.317

The ratio of relative changes from t1 is the relative change in the fluticasone group divided by the relative change in the placebo group. A ratio of 1 corresponds to no differential effect between fluticasone and placebo, a ratio below 1 corresponds to a beneficial blunting effect of fluticasone on allergen-induced IgE levels. 95% CI low and 95% CI up indicate the lower or upper bound of a 95% confidence interval, respectively.

### Rises of allergen-specific antibody isotypes other than IgE induced by nasal provocation were only moderate

**Fig 4:**
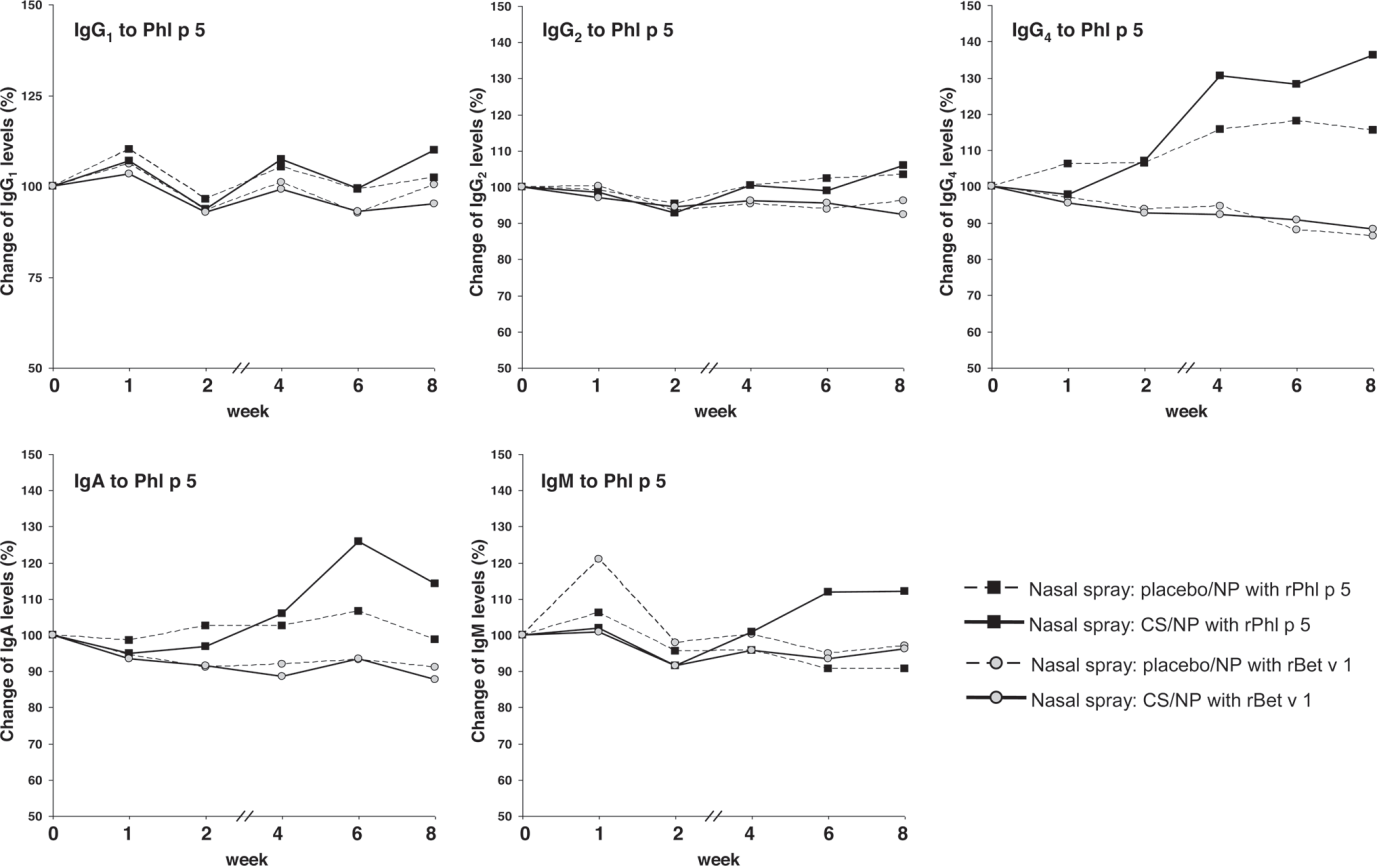
Development of Phl p 5-specific antibody responses. Percentage changes of Phl p 5-specific IgG_1_, IgG_2_, IgG_4_, IgA and IgM antibody levels (y-axes) at the day of the first nasal provocation (0) and thereafter (x-axes). Uninterrupted lines: Patients with steroid spray; dotted lines: Patients with placebo spray; black squares: provocation with rPhl p 5; grey dots: provocation with rBet v 1.

**Fig 5:**
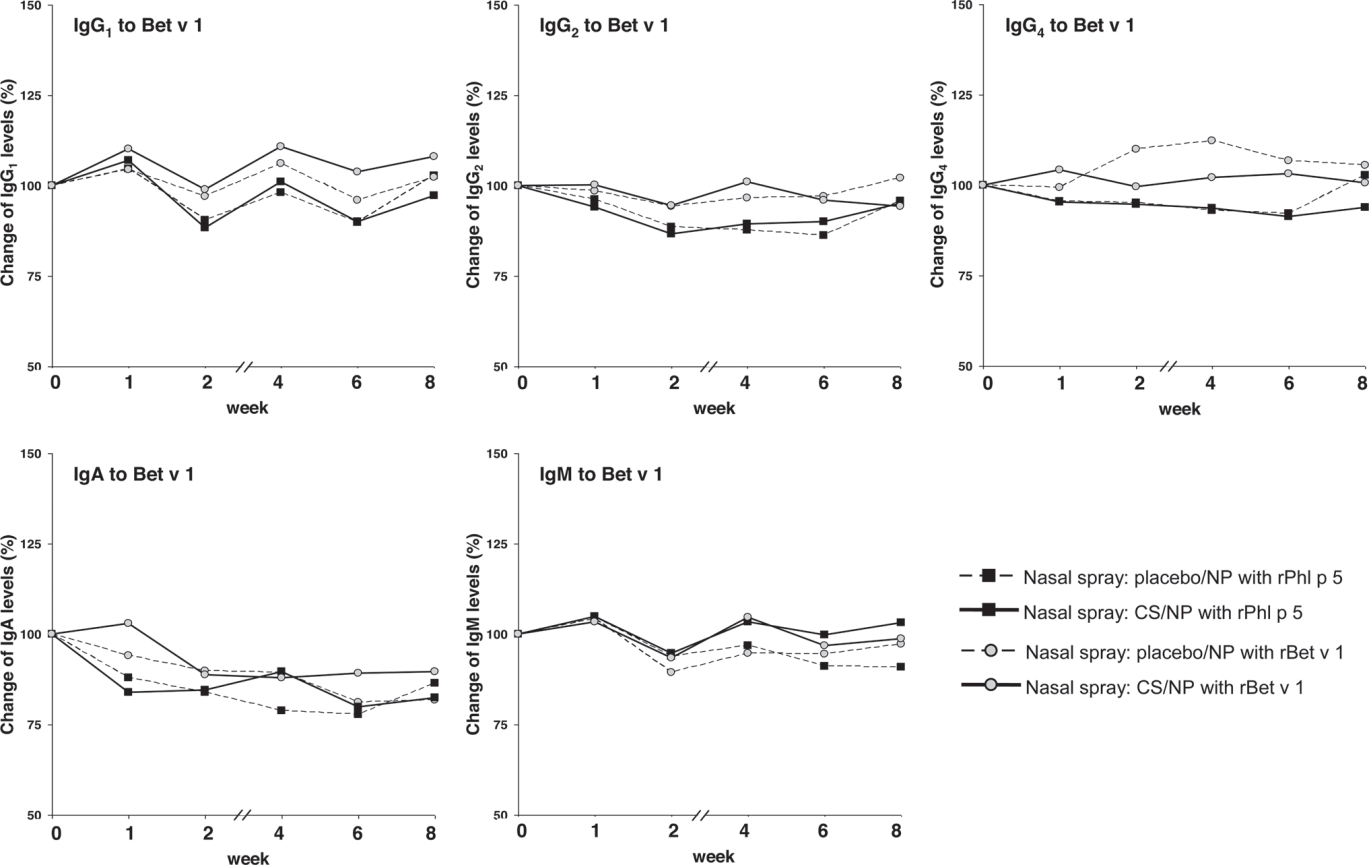
Development of Bet v 1-specific antibody responses. Percentage changes of Bet v 1-specific IgG_1_, IgG_2_, IgG_4_, IgA and IgM antibody levels (y-axes) at the day of the first nasal provocation (0) and thereafter (x-axes). Uninterrupted lines: Patients with steroid spray; dotted lines: Patients with placebo spray; black squares: provocation with rPhl p 5; grey dots: provocation with rBet v 1.

Rises of allergen-specific antibody isotypes/subclasses other than IgE were observed for Phl p 5-specific IgG_4_ levels in both placebo- and INCS-treated subjects who had received rPhl p 5 intranasally (p<0.001, difference between placebo and INCS-treatment: n.s., Fig. 4). However, the statistical significance of this result should be regarded with caution because of the small effect size and of the high number of secondary analyses that were performed. Also a small induction of Phl p 5-specific IgA levels was noted in the placebo group after nasal exposure to Phl p 5 (Fig. 4, n.s.). IgG_4_ levels to Bet v 1 and IgG_1–3_, IgA and IgM levels were very low in most subjects before provocation and did not change substantially during the weeks after provocation (Fig. 5).

## Discussion

Increases of allergen-specific IgE production in allergic patients after nasal or respiratory allergen contact may have unfavourable effects for several reasons. In fact it has been demonstrated that higher levels of allergen-specific IgE caused increased clinical sensitivity to the culprit allergens [7–12]. Furthermore, increases of IgE levels have been shown to prolong the survival of and to up-regulate the expression of Fcε receptors on mast cells, basophils and antigen presenting cells [5]. The latter leads to enhanced degranulation and release of inflammatory mediators, proteases and pro-inflammatory cytokines from mast cells and basophils [38] and to increased IgE-facilitated antigen presentation to T cells and thus to their activation [2, 3].

Therapies such as SIT which can reduce allergen-specific IgE production offer the advantage that they modulate the course of allergic disease in addition to the reduction of allergic symptoms [16–19]. In fact, it has been demonstrated that SIT can prevent the transition from mild (i.e., rhinitis) to severe (i.e., asthma) manifestations [39] and that SIT has long-term effects even after discontinuation [40].

The effects of other frequently used forms of anti-allergic treatment on allergen-specific IgE antibody production have not yet been studied in defined experimental model systems. INCS are a first line of treatment for seasonal and perennial allergic rhinitis [41] as well as for other chronic inflammatory diseases of the nose and paranasal sinuses (e.g., nasal polyps, chronic sinusitis) [42]. They seem to provide excellent control of local inflammatory conditions and are therefore widely used in adult as well as paediatric patients [43, 44]. In asthmatic patients, it has been shown that early and sustained treatment with corticosteroids may even prevent some of the irreversible airway dysfunction [45–46]. Since steroids have mainly an effect on chronic and late phase allergic inflammation but not on acute IgE-mediated mast cell degranulation, it was not unexpected that we did not find statistically significant differences between the symptoms and nasal patency in the placebo-treated and steroid-treated subjects, which was measured shortly after nasal provocation. Steroid-treated subjects recovered significantly more quickly after provocation, indicating an effect of nasal steroids on the late phase response. Furthermore, there was a trend towards less severe reduction of nasal flow after provocation in patients treated with INCS. It may thus be speculated that while the beneficial effect of steroid treatment was not measurable immediately after an acute single provocation, it should be much more obvious during prolonged allergen contact.

It has been shown earlier in an experimental model of allergic sensitization in rhesus monkeys and in clinical studies in ragweed allergic patients that the application of steroids may prevent boosts of IgE production following allergen contact [27, 47].

We therefore felt that it is important to investigate in a well-controlled clinical study utilizing defined and clinically relevant allergen molecules, whether a frequently used INCS, i.e., nasal fluticasone propionate, has effects on boosts of IgE production induced by nasal allergen contact.

Although there is considerable evidence for local IgE production in the nasal mucosa [48], we found that even a two week pre-treatment before allergen provocation, followed by a two week treatment after provocation with fluticasone propionate compared to placebo had no significant effects on the boosts of allergen-specific IgE production. Similar results were obtained for two different pollen allergens, the highly immunogenic major grass pollen allergen Phl p 5 and the less immunogenic major birch pollen allergen, Bet v 1 [49].

There are at least two explanations for the discrepancy between our study and earlier studies describing a reduction of allergen-specific IgE levels upon administration of corticosteroids. In the rhesus monkey study [47] triamcinolone acetonide was given systemically and, in the ragweed trial, steroids (i.e., beclomethasone propionate, triamcinolone acetonide) with a high bioavailability of more than 40% and a systemic immunosuppressive effect were used, whereas the bioavailability of nasal fluticasone propionate used in our study is less than 1% [50].

In a recent study we found that boosts of allergen-specific IgE production can only be obtained with intact folded allergen which is capable of interacting with the B cell receptor but not by unfolded allergen derivatives which contain the allergen-specific T cell epitopes but cannot bind to the B cell receptor [33]. The failure of steroids to suppress allergen-specific IgE production may therefore be explained by their inability to influence secondary IgE production in memory B cells and plasma cells.

In summary, we found that intranasal application of 200 μg of fluticasone propionate over 4 weeks, a regimen which corresponds to the recommended treatment for allergic patients, had no blunting effect on the boost of systemic allergen-specific IgE production following nasal provocation with 1.6 μg of Phl p 5 or Bet v 1, two major pollen allergens, administered on two consecutive days, and did not affect the production of allergen-specific IgE antibodies. Thus, other effects of intranasal corticosteroids, like a reduction of Th2-cytokine producing cells [25] as well as decreased eosinophil and mast cell numbers [51] may account for the beneficial clinical effect of intranasal corticosteroids.

One limitation of our study is that nasal provocation exposed patients to relatively high doses of allergen, corresponding to a day´s worth of allergen exposure, in a short time. We cannot exclude that INCS affect systemic IgE levels differently in patients who encounter lower doses of allergens over a more prolonged period of time. Therefore, it will certainly be interesting to study the effects of INCS on allergen-specific IgE production and subsequent allergen sensitivity in large groups of patients exposed to allergen in a pollen chamber or under conditions of natural allergen exposure. Furthermore, it will be interesting to study local allergen-specific antibody production in nasal secretions of allergic patients after allergen contact. However, the results from our trial indicate that unlike SIT, INCS cannot down-modulate allergen-specific IgE production.

## Supporting Information

S1 CONSORT Checklist (DOC)

S1 Protocol Study protocol submitted to the Ethics committee. (DOC)

S1 Study Protocol Amendment Amendment 1 and Amendment 2 to the study protocol, submitted to the Ethics committee during the study. (DOC)

S2 Study Protocol Amendment Amendment 1 and Amendment 2 to the study protocol, submitted to the Ethics committee during the study. (DOC)

S1 Table (PDF)

## References

[1] Bischoff SC (2007) Role of mast cells in allergic and non-allergic immune responses: comparison of human and murine data. Nat Rev Immunol 7: 93–104. 17259966 10.1038/nri2018

[2] van Neerven RJ, Knol EF, Ejrnaes A, Würtzen PA (2006) IgE-mediated allergen presentation and blocking antibodies: regulation of T-cell activation in allergy. Int Arch Allergy Immunol 141: 119–129. 16864979 10.1159/000094714

[3] Bieber T (1997) FcεRI-expressing antigen-presenting cells: new players in the atopic game. Immunol Today 18: 311–313. 9238831 10.1016/s0167-5699(97)01046-3

[4] Kawakami T, Galli SJ (2002) Regulation of mast-cell and basophil function and survival by IgE. Nat Rev Immunol 2: 773–786. 12360215 10.1038/nri914

[5] Saini SS, MacGlashan D (2002) How IgE upregulates the allergic response. Curr Opin Immunol 14: 694–697. 12413517 10.1016/s0952-7915(02)00404-1

[6] Gieras A, Focke-Tejkl M, Ball T, Verdino P, Hartl A, et al (2007) Molecular determinants of allergen-induced effector cell degranulation. J Allergy Clin Immunol 119: 384–390. 17291855 10.1016/j.jaci.2006.09.034

[7] Skov-Stahl P, Norh S, Weeke B (1977) Basophil histamine release in patients with hay fever. Results compared with specific IgE and total IgE during immunotherapy. Clin Exp Immunol 27: 432–439. 67913 PMC1540951

[8] Fennerty AG, Jones KP, Fifield R, Davies BH (1987) Challenge tests and specific IgE in hay fever sufferers. Clin Allergy 17: 365–372. 3621553 10.1111/j.1365-2222.1987.tb02026.x

[9] Southam DS, Ellis R, Wattie J, Inman MD (2007) Components of airway hyperresponsiveness and their associations with inflammation and remodelling in mice. J Allergy Clin Immunol 119: 848–854. 17321577 10.1016/j.jaci.2006.12.623

[10] Dente FL, Bacci E, Di Franco A, Giannini D, Vagaggini B, et al (2000) Natural exposure to pollen reduces the threshold but does not change the pattern of response to the allergen in allergic subjects. Respir Med 94: 1073–1078. 11127494 10.1053/rmed.2000.0907

[11] Niederberger V, Ring J, Rakoski J, Jäger S, Spitzauer S, et al (2007) Antigens Drive Memory IgE Responses in Human Allergy via the Nasal Mucosa. Int Arch Allergy Immunol 142: 133–144. 17057411 10.1159/000096439

[12] Skamstrup Hansen K, Vieths S, Vestergaard H, Skov PS, Bindslev-Jensen C, et al (2001) Seasonal variation in food allergy to apples. J Chromatogr B Biomed Sci Appl 756: 19–32. 11419712 10.1016/s0378-4347(01)00068-8

[13] Peroni DG, Boner AL, Vallone G, Antolini I, Warner JO (1994) Effective allergen avoidance at high altitude reduces allergen-induced bronchial hyperresponsiveness. Am J Respir Crit Care Med 149: 1442–1446. 8004296 10.1164/ajrccm.149.6.8004296

[14] Henderson LL, Larson JB, Gleich GJ (1975) Maximal rise in IgE antibody following ragweed pollination season. J Allergy Clin Immunol 55: 10–15. 45925 10.1016/s0091-6749(75)80003-0

[15] Naclerio RM, Adkinson NF Jr, Moylan B, Baroody FM, Proud D, et al (1997) Nasal provocation with allergen induces a secondary serum IgE antibody response. J Allergy Clin Immunol 100: 505–510. 9338545 10.1016/s0091-6749(97)70143-x

[16] Mothes N, Heinzkill M, Drachenberg KJ, Sperr WR, Krauth MT, et al (2003) Allergen-specific immunotherapy with a monophosphoryl lipid A-adjuvanted vaccine: reduced seasonally boosted immunoglobulin E production and inhibition of basophil histamine release by therapy-induced blocking antibodies. Clin Exp Allergy 33: 1198–1208. 12956739 10.1046/j.1365-2222.2003.01699.x

[17] Niederberger V, Horak F, Vrtala S, Spitzauer S, Krauth MT, et al (2004) Vaccination with genetically engineered allergens prevents progression of allergic disease. Proc Natl Acad Sci U S A 101 Suppl 2: 14677–14682 15310844 10.1073/pnas.0404735101 PMC521981

[18] Creticos PS, Schroeder JT, Hamilton RG, Balcer-Whaley SL, Khattignavong AP, et al (2006) Immunotherapy with a ragweed-toll-like receptor 9 agonist vaccine for allergic rhinitis. N Engl J Med 355:1445–1455 17021320 10.1056/NEJMoa052916

[19] Gadermaier E, Staikuniene J, Scheiblhofer S, Thalhamer J, Kundi M (2011) Recombinant allergen-based monitoring of antibody responses during injection grass pollen immunotherapy and after 5 years of discontinuation. Allergy 66:1174–1182 10.1111/j.1398-9995.2011.02592.x 21480924

[20] Hemady Z, Gellis S, Chambers M, Rocklin RE (1985) Effect of dexamethasone on de novo IgE synthesis by human blood lymphocytes. J Allergy Clin Immunol 75: 304–312. 3918092 10.1016/0091-6749(85)90062-4

[21] Fischer A, König W (1990) Regulation of CD23 expression, soluble CD23 release and immunoglobulin synthesis of peripheral blood lymphocytes by glucocorticoids. Immunology 71: 473–479. 2149121 PMC1384865

[22] Bohle B, Willheim M, Baier K, Stadler B, Spitzauer S, et al (1995) Hydrocortisone enhances total IgE levels—but not the synthesis of allergen- specific IgE—in a monocyte-dependent manner. Clin Exp Immunol 101: 474–479. 7664494 10.1111/j.1365-2249.1995.tb03137.x PMC1553237

[23] Cho YJ, Hong SJ, Moon HB (2000) Hydrocortisone enhances allergen-specific IgE production by peripheral blood mononuclear cells from atopic patients with high serum allergen-specific IgE levels. Clin Exp Allergy 30: 1576–1581. 11069566 10.1046/j.1365-2222.2000.00991.x

[24] Zieg G, Lack G, Harbeck RJ, Gelfand EW, Leung DY (1994) In vivo effects of glucocorticoids on IgE production. J Allergy Clin Immunol 94: 222–230. 8064074 10.1016/0091-6749(94)90044-2

[25] Durham SR, Gould HJ, Thienes CP, Jacobson MR, Masuyama K, et al (1997) Expression of epsilon germ-line gene transcripts and mRNA for the epsilon heavy chain of IgE in nasal B cells and the effects of topical corticosteroid. Eur J Immunol 27: 2899–2906. 9394816 10.1002/eji.1830271123

[26] Henderson LL, Larson JB, Gleich GJ (1973) Effect of corticosteroids on seasonal increases in IgE antibody. J Allergy Clin Immunol 52: 352 4752933 10.1016/0091-6749(73)90094-8

[27] Naclerio RM, Adkinson NF Jr, Creticos PS, Baroody FM, Hamilton RG, et al (1993) Intranasal steroids inhibit seasonal increases in ragweed-specific immunoglobulin E antibodies. J Allergy Clin Immunol 92: 717–721. 8227863 10.1016/0091-6749(93)90015-8

[28] Pullerits T, Praks L, Sjöstrand M, Rak S, Skoogh BE, et al (1997) An intranasal glucocorticoid inhibits the increase of specific IgE initiated during birch pollen season. J Allergy Clin Immunol 100: 601–605. 9389288 10.1016/s0091-6749(97)70162-3

[29] Laffer S, Valenta R, Vrtala S, Susani M, van Ree R, et al (1994) Complementary DNA cloning of the major allergen Phl p I from timothy grass (Phleum pratense); recombinant Phl p I inhibits IgE binding ot group I allergens from eight different grass species. J Allergy Clin Immunol 94: 689–698. 7930302 10.1016/0091-6749(94)90176-7

[30] Vrtala S, Sperr WR, Reimitzer I, van Ree R, Laffer S, et al (1993) cDNA cloning of a major allergen from timothy grass (Phleum pratense) pollen; characterization of the recombinant Phl p V allergen. J Immunol 151: 4773–4781. 7691956

[31] Breiteneder H, Pettenburger K, Bito A, Valenta R, Kraft D, et al (1989) The gene coding for the major birch pollen allergen Bet v 1, is highly homologous to a pea disease resistance response gene. EMBO J 8: 1935–1938. 2571499 10.1002/j.1460-2075.1989.tb03597.x PMC401053

[32] Durham SR, Walker SM, Varga EM, Jacobson MR, O'Brien F (1999) Long-term clinical efficacy of grass-pollen immunotherapy. N Engl J Med 341: 468–75. 10441602 10.1056/NEJM199908123410702

[33] Egger C, Horak F, Vrtala S, Valenta R, Niederberger V (2010) Nasal application of rBet v 1 or no-IgE-reactive T-cell epitope-containing rBet v 1 fragments has different effects on systemic allergen-specific antibody responses. J Allergy Clin Immunol 126:1312–1315. 10.1016/j.jaci.2010.06.008 20673979

[34] Schäppi GF, Taylor PE, Pain MC, Cameron PA, Dent AW, et al (1999) Concentrations of major grass group 5 allergens in pollen grains and atmospheric particles: implications for hay fever and allergic asthma sufferers sensitized to grass pollen allergens. Clin Exp Allergy 29: 633–641. 10231323 10.1046/j.1365-2222.1999.00567.x

[35] Andersson K, Lidholm J (2003) Characteristics and immunobiology of grass pollen allergens. Int Arch Allergy Immunol 130: 87–107. 12673063 10.1159/000069013

[36] Mösges R, Lehmacher W, Pasch N, Vent J (2009) Assessment of the antiobstructive effect of fexofenadine on nasal allergy challenge in patients with seasonal allergic rhinitis. Asian Pac J Allergy Immunol 27:181–90. 20232572

[37] Hanf G, Noga O, O'Connor A, Kunkel G (2004) Omalizumab inhibits allergen challenge-induced nasal response. Eur Respir J 23:414–8. 15065831 10.1183/09031936.04.00024504

[38] Kawakami T, Galli SJ (2002) Regulation of mast-cell and basophil function and survival by IgE. Nat Rev Immmunol 2: 773–786. 12360215 10.1038/nri914

[39] Möller C, Dreborg S, Ferdousi HA, Halken S, Høst A, et al (2002) Pollen immunotherapy reduces the development of asthma in children with seasonal rhinoconjunctivitis (the PAT-study). J Allergy Clin Immunol 109: 251–256 11842293 10.1067/mai.2002.121317

[40] Durham SR, Walker SM, Varga EM, Jacobson MR, O'Brien F, et al (1999) Long-term clinical efficacy of grass-pollen immunotherapy. N Engl J Med 341: 468–475 10441602 10.1056/NEJM199908123410702

[41] Bousquet J, Khaltaev N, Cruz AA, Denburg J, Fokkens WJ, et al (2008) Allergic Rhinitis and its Impact on Asthma (ARIA) 2008 update (in collaboration with the World Health Organization, GA(2)LEN and AllerGen). Allergy 63 Suppl 86:8–160. 10.1111/j.1398-9995.2007.01620.x 18331513

[42] Fokkens W, Lund V, Bachert C, Clement P, Hellings P, et al (2005) EAACI Position Paper on Rhinosinusits and Nasal Polyps Executive Summary. Allergy 60: 583–601. 15813802 10.1111/j.1398-9995.2005.00830.x

[43] Passalacqua G, Albano M, Canonica GW, Bachert C, Van Cauwenberge P, et al (2000) Inhaled and nasal corticosteroids: safety aspects. Allergy 55:16–33. 10696853 10.1034/j.1398-9995.2000.00370.x

[44] Agertoft L, Pedersen S (1994) Effects of long-term treatment with an inhaled corticosteroid on growth and pulmonary function in asthmatic children. Respir Med 88: 373 8036306 10.1016/0954-6111(94)90044-2

[45] Haahtela T, Jarvinen M, Kava T, Kiviranta K, Koskinen S, et al (1994) Effects of reducing or discontinuing inhaled budesonide in patients with mild asthma. N Engl J Med 331: 700 8058076 10.1056/NEJM199409153311103

[46] Selroos O, Pietinalho A, Lofroos AB, Riska H (1995) Effect of early vs late intervention with inhaled corticosteoids in asthma. Chest 108: 1228 7587421 10.1378/chest.108.5.1228

[47] Ferreira FD, Mayer P, Sperr WR, Valent P, Seiberler S, et al (1996) Induction of IgE antibodies with predefined specificity in rhesus monkeys with recombinant birch pollen allergens, Bet v 1 and Bet v 2. J Allergy Clin Immunol 97: 95–103. 8568143 10.1016/s0091-6749(96)70287-7

[48] Durham SR, Gould HJ, Hamid QA (1997) Local IgE production in nasal allergy. Int Arch Allergy Immunol 113: 128–130 9130501 10.1159/000237525

[49] Vrtala S, Ball T, Spitzauer S, Pandjaitan B, Suphioglu C, et al (1998) Immunization with purified natural and recombinant allergens induces mouse IgG1 antibodies that recognize similar epitopes as human IgE and inhibit the human IgE-allergen interaction and allergen-induced basophil degranulation. J Immunol 15: 6137–6144. 9637531

[50] Derendof H, Meltzer EO (2008) Molecular and clinical pharmacology of intranasal corticosteroids: clinical and therapeutic implications. Allergy 63: 1292–1300. 10.1111/j.1398-9995.2008.01750.x 18782107

[51] Rak S, Jacobson MR, Sudderick RM, Masuyama K, Juliusson S, et al (2006) Influence of prolonged treatment with topical corticosteroid (fluticasone propionate) on early and late phase nasal responses and cellular infiltration in the nasal mucosa after allergen challenge. Clin Exp Allergy 24:930–939. 10.1111/j.1365-2222.1994.tb02724.x 7842362

