# Supplementary material for: Effects of Nasal Corticosteroids on Boosts of Systemic Allergen-Specific IgE Production Induced by Nasal Allergen Exposure
Source: PLoS One. 2015 Feb 23;10(2):e0114991. doi: 10.1371/journal.pone.0114991 (PMC4338223; doi:10.1371/journal.pone.0114991)
Supplement: S1 Protocol — (DOC) [file pone.0114991.s002.doc]

The effect of intranasal corticosteroids on the immune response following nasal allergen challenge in patients suffering from seasonal allergic rhinitis

Protocol authors:

Dr. Cornelia Egger^1^

ao.Univ. Prof. Dr. Verena Niederberger^3^

o.Univ. Prof. Dr. Rudolf Valenta^1^

Principal investigator:

ao.Univ. Prof. Dr. Friedrich Horak^2,3^

Co-investigator:

Dr. Cornelia Egger^1^

^1^ Abteilung für Immunpathologie, Institut für Pathophysiologie, Zentrum für Physiologie und Pathophysiologie, Medizinische Universität Wien

Währinger Gürtel 18-20, A-1090 Wien, Österreich

Tel: +43-1-40400-5101

Fax: +43-1-40400-5130

^2^ Allergiezentrum Wien West

Hütteldorferstraße 46, A-1150 Wien

Tel: +43-1-9824121

Fax:: +43-1-9824121-4

^3^ Universitätsklinik für Hals-Nasen-Ohren-Heilkunde, AKH, Medizinische Universität Wien

Währinger Gürtel 18-20, A-1090 Wien, Österreich

Tel: +43-1-40400-3336

Fax: +43-1-40400-2998

Table of contents

[1 Introduction 6](#__RefHeading___Toc113961756)

[1.1 Background 6](#__RefHeading___Toc113961757)

[1.2 Rationale of the study 9](#__RefHeading___Toc113961758)

[2 Study objectives 9](#__RefHeading___Toc113961759)

[2.1 Primary objective 9](#__RefHeading___Toc113961760)

[2.2 Secondary objectives 10](#__RefHeading___Toc113961761)

[3 Overall design and plan of the study 10](#__RefHeading___Toc113961762)

[3.1 Overview 10](#__RefHeading___Toc113961763)

[3.2 Endpoints 12](#__RefHeading___Toc113961764)

[3.2.1 Primary endpoint 12](#__RefHeading___Toc113961765)

[3.2.2 Secondary endpoints 12](#__RefHeading___Toc113961766)

[3.3 Sample size considerations 12](#__RefHeading___Toc113961767)

[4 Study population 13](#__RefHeading___Toc113961768)

[4.1 Number of subjects 13](#__RefHeading___Toc113961769)

[4.2 Patient recruitment 13](#__RefHeading___Toc113961770)

[4.3 Eligibility criteria 13](#__RefHeading___Toc113961771)

[4.3.1 Inclusion criteria 13](#__RefHeading___Toc113961772)

[4.3.2 Exclusion criteria 13](#__RefHeading___Toc113961773)

[4.4 Subject identification and de-personalization 15](#__RefHeading___Toc113961774)

[4.5 Subject withdrawal 15](#__RefHeading___Toc113961775)

[5 Study procedures 15](#__RefHeading___Toc113961776)

[5.1 Case history 15](#__RefHeading___Toc113961777)

[5.1.1 Symptom score 15](#__RefHeading___Toc113961778)

[5.2 Physical examination 16](#__RefHeading___Toc113961779)

[5.2.1 Auscultation 16](#__RefHeading___Toc113961780)

[5.2.2 Anterior rhinoscopy 16](#__RefHeading___Toc113961781)

[5.3 FEV1 measurement 16](#__RefHeading___Toc113961782)

[5.4 Skin prick test 16](#__RefHeading___Toc113961783)

[5.4.1 Method 16](#__RefHeading___Toc113961784)

[5.4.2 Procedure 17](#__RefHeading___Toc113961785)

[5.4.3 Test solutions 17](#__RefHeading___Toc113961786)

[5.4.4 SPT in study conduct 17](#__RefHeading___Toc113961787)

[5.4.5 Safety precautions 18](#__RefHeading___Toc113961788)

[5.5 Blood sampling 18](#__RefHeading___Toc113961789)

[5.5.1 Measurement of total IgE 18](#__RefHeading___Toc113961790)

[5.5.2 Measurement of specific IgE 18](#__RefHeading___Toc113961791)

[5.5.3 Dot Blot 19](#__RefHeading___Toc113961792)

[5.5.4 CD203c and histamine release assay 19](#__RefHeading___Toc113961793)

[5.6 Pregnancy testing 19](#__RefHeading___Toc113961794)

[5.7 Nasal provocation and rhinomanometry 19](#__RefHeading___Toc113961795)

[5.7.1 Method 19](#__RefHeading___Toc113961796)

[5.7.2 Procedure 20](#__RefHeading___Toc113961797)

[5.7.3 Symptom score 20](#__RefHeading___Toc113961798)

[5.7.4 Test solutions 21](#__RefHeading___Toc113961799)

[5.7.5 NPT in study conduct 21](#__RefHeading___Toc113961800)

[5.7.6 Safety precautions 21](#__RefHeading___Toc113961801)

[5.8 Statistical analysis 21](#__RefHeading___Toc113961802)

[6 Investigational products and study medication 22](#__RefHeading___Toc113961803)

[6.1 Recombinant allergens, recombinant allergen fragments and control solutions 22](#__RefHeading___Toc113961804)

[6.1.1 Recombinant Phl p 5 and Bet v 1 22](#__RefHeading___Toc113961805)

[6.1.2 Recombinant Bet v 1 fragments F1 and F2 22](#__RefHeading___Toc113961806)

[6.1.3 Control solutions 22](#__RefHeading___Toc113961807)

[6.2 Study medication 23](#__RefHeading___Toc113961808)

[6.2.1 Study drug – packaging, labelling, randomization, and storage 23](#__RefHeading___Toc113961809)

[6.2.2 Concomitant medication 24](#__RefHeading___Toc113961810)

[7 Data handling procedure 24](#__RefHeading___Toc113961811)

[8 Adverse events 25](#__RefHeading___Toc113961812)

[8.1 Definition of serious and non-serious adverse events 25](#__RefHeading___Toc113961813)

[8.2 Assessment of severity of AE 25](#__RefHeading___Toc113961814)

[8.3 Reporting/documentation of adverse events 26](#__RefHeading___Toc113961815)

[8.3.1 Reporting/documentation of serious adverse events 26](#__RefHeading___Toc113961816)

[8.3.2 Reporting/documentation of non-serious adverse events 26](#__RefHeading___Toc113961817)

[9 Ethical and legal aspects 26](#__RefHeading___Toc113961818)

[9.1 Declaration of Helsinki 26](#__RefHeading___Toc113961819)

[9.2 Informed consent of subject 27](#__RefHeading___Toc113961820)

[9.3 Acknowledgement / approval of the study 27](#__RefHeading___Toc113961821)

[9.4 Insurance 27](#__RefHeading___Toc113961822)

[9.5 Confidentiality 27](#__RefHeading___Toc113961823)

[10 Documentation and use of study findings 27](#__RefHeading___Toc113961824)

[10.1 Documentation of study findings 27](#__RefHeading___Toc113961825)

[10.2 Use of study findings 28](#__RefHeading___Toc113961826)

[11 Protocol amendments 28](#__RefHeading___Toc113961827)

[12 References 29](#__RefHeading___Toc113961828)

[13 Appendices 33](#__RefHeading___Toc113961829)

Abbreviations

APC antigen presenting cells

AR allergic rhinitis

CRF case record form

INCS intranasal corticosteroids

mAb monoclonal antibody

NPT nasal provocation test(ing)

SPT skin prick test(ing)

# Introduction

## Background

Allergic rhinitis (AR) is a common manifestation of Type I allergy, an IgE-mediated hypersensitivity disease affecting more than 25% of the population (Wüthrich, 1995). Diagnosis is based upon case history, *in vivo* (skin tests, provocation tests) and *in vitro* (specific IgE) tests. Therapeutic options of allergic rhinitis comprise allergen avoidance, immunotherapy and symptomatic treatment with local or systemic antihistamines, local or systemic corticosteroids, cromones, decongestants, and antileukotrienes (Members of the workshop, 2004).

In seasonal AR annual fluctuations in detectable specific serum IgE levels are long known, reaching a maximum after the pollen season with subsequent reduction until exposure reoccurs (Sadan, 1969; Yunginger1973). An allergen exposure-induced IgE boost has also been observed after nasal provocation (Naclerio, 1997) but not after skin prick testing (SPT). Furthermore, it has been shown that the rise of allergen-specific serum IgE results in an increase in cutaneous sensitivity and that skin test results reflect immediate type respiratory sensitivity (Niederberger, 2001; Niederberger, unpublished observations).

Conceivable immunological mechanisms leading to the rise of allergen specific IgE after allergen contact via the respiratory mucous membranes are, on the one hand, the IgE epitope-dependent activation of allergen specific B-memory cells and subsequent IgE production in the nasal mucosa or, on the other hand, allergen uptake and processing by mucosal antigen presenting cells (APC) which then migrate to the draining lymph nodes where activation of B-cells and IgE production occur.

Nasal provocation testing (NPT) is a useful tool to study the reaction of the nasal mucosa to inhalants under standardized conditions and a standard diagnostic procedure in clinical routine. It allows to discriminate between clinically irrelevant sensitization and true allergy. In rare cases, NPT is the only means available to provide evidence for allergic sensitization because local IgE production in nasal mucosa without systemically elevated IgE levels can account for AR (Huggins KG, 1975). Active anterior rhinomanometry represents a sensitive method to assess objective clinical parameters of nasal obstruction (nasal flow, nasal resistance and flow decrease) (Bachmann, 1982; Clement, 1984; Riechelmann, 2002).

Until now, crude allergen extracts which not only contain the disease-eliciting molecules but also other allergenic and non-allergenic compounds are used for SPT, NPT, and *in vitro* tests to diagnose allergy in daily clinical practice. However, during the last decade, purified natural or recombinant allergen molecules representing the epitope complexity of the natural allergen have been obtained for most allergen sources (Valenta, 1999). To date, several recombinant allergens are already commercially available for routine *in vitro* use and complement allergen extracts for allergy diagnosis.

Among these recombinant allergens, rPhl p 5 and rBet v 1 represent the recombinant counterparts of natural Phl p 5, a major grass pollen allergen, and natural Bet v 1, the major birch pollen allergen. In addition to their *in vitro* use, both allergens have been used for *in vivo* testing, i.e. skin testing, nasal, conjunctival, and even bronchial provocation testing, in numerous studies (Godnic-Cvar, 1997; Heiss, 1999; Arquint, 1999; Van Hage-Hamsten, 2002; Niederberger, 2001; Arzneimittelbeiratsgutachten AG700). Thereby, it has been shown that they exhibit the same immunological properties as their natural counterparts without giving rise to increased safety concerns (Van Hage-Hamsten, 2004).

In parallel, hypoallergenic allergen derivatives have been engineered as candidates for immunotherapy, the only causative treatment of allergy, in order to limit side effects while conserving structures encountering for its protective effects.

For Bet v 1, a hypoallergenic derivative was obtained by dividing the molecule into two halves. These two fragments F1 (amino acids 1-74) and F2 (amino acids 75-160) were extensively evaluated in *in vitro* and *in vivo* experiments and recently entered immunotherapy studies. They have proved to show a marked reduction in IgE binding capacity without loosing T-cell stimulating properties and in clinical studies skin test reactivity to the fragments was more than 100-fold reduced as compared to native Bet v 1 (Vrtala, 1997; Van Hage-Hamsten 2002; Niederberger, 2004).

Intranasal corticosteroids (INCS) are regarded as a highly effective, first line treatment for patients suffering from AR with moderate to severe and/or persistent symptoms. They should be given regularly and, in severe cases, therapy may be started before the beginning of the pollen season (Van Cauwenberge, 2000). INCS downregulate allergic inflammation in the nasal mucosa via a broad range of effects on transcription factors and cytokine production (e.g. IL-4, 5, 6, 8, 13, ICAM-1, VCAM-1) in numerous cells (epithelial cells, lymphocytes, eosinophils, basophils, mast cells, langerhans cells) and reduction of inflammatory cell infiltration. However, although a connection between chronic inflammation and symptoms has been established in allergic rhinitis, it is not clear how the anti-inflammatory effects of INCS are associated with their ability to reduce early-phase symptoms (Mygind, 2001). Side effects of INCS are mild and confined to the nasal mucosa in almost all cases (Passalacqua, 2000).

Mometasone furoate is considered the most potent INCS presently on the market. At the same time intranasal mometasone has an extremely low rate of systemic side effects. In studies on the pharmacodynamic properties of oral and/or intranasal mometasone furoate no suppression of the hypothalamic-pituitary-adrenal axis function was found. Its systemic absorption after intranasal administration is extremely low with a bioavailibility of ≤0.1%. It undergoes extensive hepatic metabolism, so that systemic activity from the swallowed portion is negligible. In AR, the recommended therapeutic dose of aqueous mometasone furoate is 200ug administered once daily as an intranasal spray (100ug to each nostril). The dose may be doubled if control of symptoms is insufficient. Improvement of nasal symptoms starts after several hours and reaches its maximum after 7 to 14 days of continuous treatment. In clinical trials with mometasone furoate, the overall incidence of adverse events was similar to placebo. Observed side effects were usually mild to moderate in severity and of short duration. The only side effect which was observed more frequently with mometasone than with placebo was mild epistaxis. There is no association of adverse tissue changes (e.g. atrophy) with the use of the drug (Onrust, 1998; Szefler, 2001).

Previous studies (Welsh, 1987; Naclerio RM, 1993; Pullerits T 1997) investigating the effect of intranasal corticosteroids on the humoral immune response to seasonal allergen exposure have yielded controversial results.

Welsh and colleagues categorized 120 patients allergic to ragweed pollen into four groups based on type of treatment (placebo, cromolyn, flunisolide, or beclomethasone) and compared ragweed specific serum IgE levels pre- and postseasonally. All intranasal topical treatments failed to reduce the seasonal increase of ragweed-specific IgE antibody levels. In contrast, Naclerio *et al*. (1993) found that treatment with INCS decreased the rise of ragweed-specific IgE in two smaller trials comparing either beclomethasone dipropionate or triamcinolone acetonide with placebo. Similar results were reported by Pullerits in 1997 in 20 birch pollen allergic patients.

In only one study, nasal provocation was used to address this question in a systematic and controlled approach (Naclerio, 1997). Patients were treated with beclomethasone dipropionate for seven days before and throughout 3 consecutive days of intranasal challenges with ragweed pollen extract. No effect of INCS on the humoral immune response was found.

However, all previous clinical studies were performed with allergen extracts containing a number of different allergens in vague amounts. To date, the availability of recombinant allergens allows the application of defined allergen amounts and to evaluate allergen-specific immune responses following allergen exposure.

## Rationale of the study

Previous studies on the effect of INCS on the humoral IgE response after allergen exposure yielded conflicting results. In this study we aim to investigate the effect of INCS on allergen exposure-induced IgE rises by the means of controlled NPT with pure recombinant allergens. NPT mimics natural allergen exposure but allows to standardize its conditions (e.g. amount, period). The use of recombinant allergens allows to investigate IgE responses specifically to the disease-eliciting antigens.

In a second part of the study, the immunological mechanism underlying the allergen induced boost of IgE production will be investigated. The use of non-IgE reactive fragments of the major birch pollen allergen Bet v 1 (Vrtala, 1997) *vs.* the complete IgE-reactive molecule will allow us to determine whether the boost of IgE production depends on IgE-mediated mechanisms or purely T-cell dependent mechanisms.

# Study objectives

## Primary objective

To evaluate the effect of mometasone furoate *vs.* placebo applied as nasal spray on the increase of allergen specific serum IgE levels following nasal exposure to recombinant allergens. Nasal sprays will be applied for four weeks starting two weeks before nasal provocation and serum IgE levels will be followed during eight weeks after nasal provocation.

## Secondary objectives

- To assess the allergen-specifity of the increased serum IgE levels after allergen exposure.
- To evaluate if allergen-specific serum IgE levels are reflected in allergen-specific skin prick test reactivity *in vivo* measured immediately before and six weeks after nasal provocation.
- To evaluate if allergen-specific serum IgE levels are reflected in allergen-specific effector cell activation *in vitro* measured by histamine release and CD203c assays immediately before and six weeks after nasal provocation.
- To determine whether the two recombinant halves of the major birch pollen allergen Bet v 1 which have shown a marked reduction in IgE binding capacity without loosing T-cell stimulating properties are able to induce the increase of allergen-specific serum IgE levels following nasal exposure.
- To determine the effect of INCS on nasal flow at NPT.

# Overall design and plan of the study

## Overview

In total, 90 patients will be included in the study on a voluntary basis. 30 mainly grass pollen and 30 mainly birch pollen allergic patients sensitized to rPhl p 5 and rBet v 1 will enter a single-center, randomized, placebo-controlled, double-blind study. In parallel, another 30 birch pollen allergic patients will be evaluated in an observational study.

**Screening.** During a first screening visit patients will be orally informed about the study protocol and will be asked to read and sign the consent form. History will be obtained, and anterior rhinoscopy, FEV1 measurement, SPT with recombinant allergens, and blood sampling (40 ml) will be performed to determine inclusion and exclusion criteria. A part of the serum sample will be directly sent to the laboratory for analysis of total IgE and specific IgE to rPhl p 5 and rBet v 1 and the rest will be stored at –20°C. In addition, in female patients, pregnancy will be excluded with a standard urine pregnancy test, which will be repeated monthly.

**Inclusion.** Patients suitable according to the inclusion and exclusion criteria will be enrolled in the study and a study number will be allotted. Patients in the double-blind, placebo-controlled part of the study will be invited to the inclusion visit.

During this visit they will receive the study medication (nasal sprays containing mometasone furoate or placebo) which will be provided by Aesca Ges.m.b.H., Traiskirchen, in an encoded manner. They will be instructed in the correct use of nasal sprays. Nasal sprays will be administered during four weeks, namely 14 days prior to and 14 days after the NPT. Patients in the observational part of the study will not receive a nasal spray. They will not have to attend this visit.

The first day of nasal spray application will be referred to as *day 1* of the study.

**Nasal provocation 1.** The third visit will take place two weeks after the beginning of administration of nasal sprays (mometasone or placebo) (*day 15*). Any adverse events will be recorded. A blood sample (40 ml serum, 20 ml heparinized blood) will be obtained. Heparinized blood will be analyzed regarding CD203c expression and basophil histamine release and sera will be stored at -20°C for future determination of baseline parameters. SPT with recombinant Phl p 5, Bet v 1 and its fragments and a first NPT with either rBet v 1 or rPhl p 5 will be performed (“nasal priming”). Selection of the allergen will be based on symptom severity as reported by the patient during seasonal pollen exposure. Patients will be monitored for at least 60 minutes after nasal allergen challenge. Patients in the observational part of the study, who will not receive a nasal spray, will be challenged with the recombinant fragments F1 and F2 of Bet v 1.

**Nasal provocation 2.** One day after the first nasal challenge (*day 16*) NPT will be repeated with the same allergen to induce the increase of serum IgE levels. Again, patients will be monitored for at least 60 minutes after nasal allergen exposure.

**Observation period.** Blood samples (40ml) will be collected on *days 23* (visit 5), *30* (visit 6), and *44* (visit 7). On *day 58* (visit 8), 60 ml blood (40 ml serum and 20 ml heparinized blood) will be taken and SPT with recombinant allergens will be repeated.

**End of study.** On *day 72* (visit 9), a final blood sample (40 ml) will be obtained and the study will be completed.

The study will be conducted between November and February, i.e. outside the birch and grass pollen season, to avoid interference by natural pollen exposure.

## Endpoints

### Primary endpoint

- change of rPhl p 5 or rBet v 1 (according to the allergen used for NPT) specific serum IgE antibody levels during 8 weeks following NPT.

### Secondary endpoints

- change of rBet v 1 or rPhl p 5 (according to the allergen not used for NPT) specific serum IgE antibody levels during 8 weeks following NPT.
- change of mean wheal diameter (skin prick test reactivity) to rPhl p 5 and rBet v 1 6 weeks after NPT compared to baseline immediately before NPT.
- change of effector cell reactivity to rBet v 1 and rPhl p 5 in histamine release and CD203c assays 6 weeks after NPT compared to baseline immediately before NPT.
- reduction of nasal flow at NPT.

## Sample size considerations

It is expected that during the eight weeks period rPhl p 5/rBet v 1 specific serum IgE antibody levels will increase after challenge up to a level of about one standard deviation above baseline (Niederberger, submitted). This increase will be prevented by INCS and will not occur in the group challenged by the fragments. Sample size is estimated based on a target significance level of 5% and a power of 80%. Applying a two factor ANOVA design with four time points and three groups, overall 19 patients per group are necessary.

# Study population

## Number of subjects

A total of 90 volunteers with a history of seasonal AR to grass and/or birch pollen will be included in the study.

## Patient recruitment

Patients will be recruited by postings in the area of the Vienna Medical University (see Appendix 2) and by telephone contact of patients who have previously consented to being notified about clinical studies.

## Eligibility criteria

### Inclusion criteria

- male or female 18-50 years of age
- moderate to severe allergic rhinitis to grass and/or birch pollen for at least two seasons according to history
- sensitization to rPhl p 5 and rBet v 1 as demonstrated by UniCAP within a range from 3.0-80 kUA/l and a positive (wheal diameter >= 3 mm larger than the negative control and half of the histamine control) SPT with rPhl p 5 and rBet v 1 at 20 µg/ml
- willingness to comply with the study protocol
- written informed consent

### Exclusion criteria

- perennial allergic rhinitis
- history of asthma necessitating treatment
- FEV1 <70% of predicted value
- abnormalities at auscultation of heart or lungs
- history of anaphylaxis
- severe atopic dermatitis
- total serum IgE >2000 kU/l
- previous or ongoing immunotherapy (subcutaneous or sublingual) to grass or birch pollen
- nasal provocation testing during the previous six month
- known allergy/intolerance to mometasone furoate or loratadine
- known allergy/intolerance to cellulose, glycerol, polysorbate, benzalkonium chloride, cornstarch, lactose
- contraindications for the use of INCS:
  - acute or chronic infections of the upper respiratory tract
  - surgery of the nose during the previous year
  - hypersensitivity to components of the drug
- contraindications for NPT
  - acute rhinosinusitis
  - acute allergic reaction of the immediate type at other organs
- nasal polyposis or significant nasal anatomical deformities
- vasomotor rhinitis
- autoimmune disease, chronic or acute infectious disease, malignancy
- severe psychological disorder
- treatment with systemic or topical (intranasal, inhaled, external) corticosteroids from 3 month prior to the study
- treatment with other immunosuppressant drugs from 6 month prior to the study
- treatment with antihistamines or disodium cromoglycate from 2 weeks prior to the study
- treatment with intranasal adrenergic drugs from 3 days prior to the study
- treatment with systemic adrenergic drugs
- treatment with psychopharmacological drugs from 2 weeks prior to the study
- cardiovascular or pulmonary disease
- contraindication for adrenaline
- participation in any other clinical trial within the previous 3 month
- pregnant, lactating or sexually active women with childbearing potential who are not using a medically accepted birth control method
- a mental condition rendering the subject unable to understand the nature, scope and possible consequences of the study, and/or evidence of an uncooperative attitude
- known alcohol or drug addiction or abuse
- unlikelihood to be able to complete the study

## Subject identification and de-personalization

All subjects enrolled must be identifiable throughout the study. The investigator will maintain a personal list of subject names and numbers to enable records to be found at a later date.

Subject numbers will be allotted to patients suitable for the study according to the inclusion and exclusion criteria. Numbers 1-30 will be used for (primarily) grass pollen allergic patients who will be exposed to rPhl p 5 at NPT and numbers 31-60 for (primarily) birch pollen allergic patients who will be exposed to rBet v 1 at NPT. Patients in the observational part of the study will receive numbers 61-90.

## Subject withdrawal

The subjects will be advised in the Informed Consent Forms that they have the right to withdraw from the study at any time without prejudice. The subject may also be withdrawn from the study at the investigator’s discretion at any time. The date of the withdrawal, the person who initiated withdrawal and the primary reason for withdrawal (e.g. adverse events, withdrawal of consent, pregnancy, violation of the treatment plan) will be recorded in the CRF.

# Study procedures

## Case history

Patients will be asked for their medical history including demographic data and concomitant medication, their allergic symptoms including intensity and duration and their allergic medication history. History will be considered suggestive of seasonal AR if rhinorrhea, nasal congestion, nasal itching or sneezing occur during birch and/or grass flowering season, i.e. form March to July in the study area.

### Symptom score

The following symptom score will grade severity of AR. Patients will be asked for nasal itch, sneezing, rhinorrhea, and nasal blockage during the previous pollen season when symptoms were worst and to grade each symptom by applying the following scale: 0, not present; 1, mild – present, no discomfort; 2, moderate – present, some discomfort, not interfering with daily life activities; 3, severe – bothersome and interfering with daily life activities or disturbing sleep. The use of medication to control symptoms will account for 2 points. AR will be considered moderate/severe if a sum score of 4 or higher is obtained.

## Physical examination

### Auscultation

Auscultation of the heart and lungs will be performed at the screening visit and only patients with normal findings will be included in the study.

### Anterior rhinoscopy

Anterior rhinoscopy will be performed once during the screening visit to exclude anatomical variances (e.g. septal deviation) or pathological changes (e.g. nasal polyps) interfering with accurate rhinomanometry. It will be performed by using a speculum with the patient seated with the head slightly back.

## FEV1 measurement

FEV1 measurements will be performed during the first visit and only patients with a FEV1 of at least 70% of the predicted value will be included in the study.

## Skin prick test

### Method

SPT is a rapid and accurate way of identifying the causative allergens in atopic individuals. Allergens are inserted into the dermis by gently pricking the skin through a drop of an allergen-containing solution with a sterile lancet. In previously sensitized individuals, IgE molecules on the surface of dermal mast cells are bridged and degranulation of the mast cells occurs. Preformed granules containing histamine are released, resulting in the appearance of a small, itchy swelling and a reddening of the skin within 15 minutes (wheal and flare-reaction). Therefore, SPT identifies the atopic state by demonstrating tissue bound IgE and represents the *in vivo* counterpart of serum specific antibody tests.

### Procedure

SPT will be performed on the volar forearms of the subjects. 20 µl aliquots of each test solution will be applied at a distance of at least 2 cm between each individual application point. Sterile prick lancets, which will be discarded after each application to avoid possible cross-contamination, will be used to gently prick the skin through the drops. After being pricked, the patient will keep his forehand in horizontal position. After 20 minutes, allergen solutions will be wiped off and margins of the wheals will be traced with a ball point pen. Transparent tape will be bonded on the irritated skin in order to transfer the outline of the wheal to the tape. The taped copies are then fixed to a SPT form with each tape being added to the corresponding allergen solution.

The surface of the wheals will be calculated by digital planimetry. For inclusion, wheals of at least 3 mm in diameter larger than the negative control and at least half of the histamine control will be regarded as positive reactions.

### Test solutions

Two recombinant allergens, the fragments F1 and F2 (mix), and 2 control solutions will be tested as described (Vrtala, 1997; Heiss, 1999; Niederberger, 2001). The recombinant allergens rBet v 1 and rPhl p 5 will be diluted in 0.9% sodium chloride solution at 1, 5, 10, and 20 µg/ml. The recombinant fragments of Bet v 1 F1 and F2 will be diluted at 0.5, 2.5, 5, and 10 µg/ml and mixed in equal amounts which will correspond to the dilutions containing the whole rBet v 1 molecule (equimolarity). Solutions for SPT will be aliquoted on a per patient and test basis before the beginning of the study. Aliquots will be stored at –20°C and thawed about to use. As controls sterile 0.9% NaCl solution (negative control) and histamine hydrochloride 10 mg/ml (positive control) will be used (Allergopharma Joachim Ganzer KG, Reinbek, Germany). They will be stored according to the manufacturer’s instruction.

### SPT in study conduct

SPT will be performed 3 times during the study (screening visit, nasal provocation visit 1 (day 15), and visit 8 (day 58)). At the screening visit a total of 5 prick tests (rBet v 1, rPhl p 5, and mix F1/F2 at 20 μg/ml, 2 controls) will be performed. At the nasal provocation visit and visit 8 a total of 27 prick tests (rBet v 1, rPhl p 5, mix F1/F2 at 1, 5, 10, and 20 μg/ml and the positive control in duplets, negative control in singlet) will be performed.

Blood samples will be obtained before SPT.

### Safety precautions

As a safety precaution, patients will be monitored for 30 minutes after testing. Although anaphylactic events are very unlikely but always have to be considered possible, a complete emergency kit will be available immediately in case of anaphylactic reactions.

## Blood sampling

Blood samples will be taken by puncture of the antecubital vein before skin testing. Maximum 60 ml per visit will be taken for the preparation of serum, basophil histamine release assays, and CD203c assays. Precisely, 40 ml blood will be obtained at visits 1, 5, 6, 7, and 9 for the preparation of serum which will be stored at –20°C until use, and 60 ml (40 ml for the preparation of serum and 20 ml heparinized blood for immediate analysis in histamine release and CD203c assays) at visits 3 and 8.

### Measurement of total IgE

Measurement of total IgE will be performed by CAP-FEIA measurements (Pharmacia, Uppsala, Sweden), representing a standard procedure in routine diagnostics.

### Measurement of specific IgE

Allergen specific antibodies in the sera will be measured by UniCAP-FEIA (Pharmacia, Uppsala, Sweden) at the beginning of the study to check inclusion criteria and at the end of the study in all collected serum samples in one assay to eliminate interassay variation. Results will be indicated in kUA/l.

### Dot Blot

To exclude IgE reactivity to the recombinant fragments F1 and F2 of Bet v 1 in the group of patients being challenged with the fragments their sera will be analysed by IgE-dot blot. For this purpose, the fragments F1 and F2 will be dotted onto nitrocellulose strips. Strips will be incubated with the sera to probe the nitrocellulose-bound proteins and bound IgE antibodies will be detected by ^125^I-labeled anti-human IgE and subsequent autoradiography (Vrtala, 1997).

### CD203c and histamine release assay

In parallel to the SPTs at the nasal provocation 1 and visit 8 (day 58) heparinized peripheral blood will be obtained for CD203c and histamine release assays. Both assays allow the demonstration of IgE-dependent effector cell activation (Hauswirth, 2002).

For CD203c assays blood aliquots will be incubated with serial dilutions of recombinant allergens (from 10^-5^ to 1 μg/ml), anti-IgE antibody (1 μg/ml) or PBS. Cells will be washed and incubated with phycoerythrin-labeled CD203c mAb to stain cells presenting the CD203c molecule on their surface as a marker for IgE-dependent cell activation. Cells will be analyzed by flow cytometry.

For histamine release assays basophils will be enriched by dextran sedimentation and exposed to recombinant allergens or anti-IgE mAb (Valenta, 1993). After incubation, cells will be centrifuged and supernatants will be analysed for histamine content by using a commercial radioimmunoassay (Immunotech).

## Pregnancy testing

In female patients, pregnancy will be excluded with a standard urine pregnancy test.

## Nasal provocation and rhinomanometry

### Method

The purpose of rhinomanometry is to measure objectively the impact of the allergen provocation on the nasal discharge. Nasal resistance is calculated by measuring the flow through the nose as well as the pressure of force required to cause the airflow.

Active anterior rhinomanometry offers a very accurate method to objectively assess clinical parameters (nasal flow, nasal resistance and nasal flow increase) of nasal obstruction. Nasal flow and nasal resistance are observed at pressure levels of 75, 150 and 300 Pa. Statistical evaluation focuses on the sum of the right and left nasal airflow (ml/s) at a pressure difference of 150 Pa (inspiration).

In active anterior rhinomanometry, the patient – wearing an airtight face mask over the mouth and nose and having the mouth closed – breathes through one nostril to evaluate the nasal flow while a sensor in the other nostril is used to measure the difference in prenasal and choanal pressure. The system used is connected to a computer. Transducer signals of transnasal airflow and pressure are amplified, digitised and shown as curve on the screen allowing on-screen control, immediate printing and saving of data.

The evaluation of the reports focuses on the sum of nasal flow (ml/s) at 150 Pa. For statistical evaluation the change of this summarised flow from the baseline is of special interest.

### Procedure

Patients will be allowed to adapt to room climate for 15 minutes before establishing baseline levels of nasal parameters without any interaction.

Then, ten minutes after applying the diluent (0.9% sodium chloride solution) to the nostril showing the higher nasal flow at baseline a second measurement will be obtained in order to rule out any non-specific hyperreactivity to the diluent *per se* (negative control and reference).

Finally, nasal provocation will be performed by applying 2 puffs of the aqueous allergen solution into the same nostril using a metered-dose nasal pump spray delivering 15µl of solution per spraying action. Patients will be asked to hold their breath during application of the allergen solution to prevent inhalation. Active anterior rhinomanometry will be performed 10 and 20 minutes after provocation.

### Symptom score

In addition to the objective parameters measured by rhinomanometry, patients will be asked to score the symptoms of blockage, secretion and itching by using a 4-point scale (0 = no symptoms; 1 = mild symptoms; 2 = moderate symptoms; 3 = severe symptoms) and the number of sneezes will be counted (0 = no sneeze; 1 = 1-5 sneezes; 2 = 6-10 sneezes; 3 > 11 sneezes) to calculate a total nasal symptom score.

### Test solutions

The recombinant allergens rPhl p 5, rBet v 1, and a mix (1:1) of the recombinant fragments will be diluted in sterile 0.9% sodium chloride solution at 50 μg/ml (25 μg/ml for each fragment), respectively, before the beginning of the study. Solutions will be aliquoted and stored at –20°C until use.

### NPT in study conduct

Nasal provocation will be performed twice on two consecutive days in the course of the study after two weeks of treatment with either mometasone furoate or placebo.

### Safety precautions

Typical reactions to nasal provocation comprise nasal itch, sneezing, nasal hypersecretion and nasal obstruction. Patients will be provided with handkerchiefs and, if necessary, with one tablet of 5 mg desloratadine at the investigator’s discretion.

To prevent inhalation during application of the allergen solution, patients will be requested to hold their breath during application of the allergen solution. Although anaphylactic events are very unlikely at NPT but always have to be considered possible, a complete emergency kit will be available immediately in case of anaphylactic reactions.

Patients will be monitored for at least 60 minutes after allergen application.

## Statistical analysis

IgE levels will be log transformed to stabilize variances and to obtain normality. Residuals of transformed variables after ANOVA will be tested by Kolmogorov-Smirnov test for deviations from normality and by Levene’s test for homogeneity of variance. A two factor ANOVA with one repeated measurements factor and one between-subjects factor will be applied. Similar analyses will be performed for secondary endpoints but as exploratory tests. Significance level for the primary endpoint is set to 5%.

# Investigational products and study medication

## Recombinant allergens, recombinant allergen fragments and control solutions

### Recombinant Phl p 5 and Bet v 1

Recombinant Phl p 5 and recombinant Bet v 1 have been previously used for skin and provocation testing in allergic patients (Godnic-Cvar, 1997; Heiss, 1999; Arquint, 1999; Van Hage-Hamsten, 2002; Van Hage-Hamsten, 2004; Arzneimittelbeiratsgutachten AG700; Niederberger, 2001).

Recombinant proteins will be reconstituted in sterile 0.9% sodium chloride solution and dilutions of 1, 5, 10, and 20 μg/ml for SPT and 50 μg/ml for NPT will be prepared and aliquoted on a per patient and test basis.

### Recombinant Bet v 1 fragments F1 and F2

The recombinant Bet v 1 fragments F1 (amino acids 1-74) and F2 (amino acids 75-160) have already been used for skin and nasal provocation testing (Van Hage-Hamsten, 2002; Niederberger, 2004; Arzneimittelbeiratsgutachten AG700). Recombinant fragments were expressed in E. coli and purified as described (Vrtala, 1997).

### Control solutions

Control solutions will comprise sodium chloride 0.9% as negative control and histamine 10 mg/ml as positive control. They will be obtained from Allergopharma, Reinbek, Germany, and stored according to the manufacturer’s instructions.

## Study medication

### Study drug – packaging, labelling, randomization, and storage

*Nasonex aquosum-Nasenspray* and placebo will be supplied by AESCA Ges.m.b.H., Traiskirchen, in an encoded manner. Labels will display numbers 1-60 and be randomized in blocks of 4. Sprays will be dispensed in ascending order from numbers 1-30 for mainly grass pollen allergic patients and numbers 31 to 60 for mainly birch pollen allergic patients. To prevent shortness of the drug, nasal sprays will be available twofold for each patient. Patients will hand-in the first package and be provided with the second package at NPT 1 (halftime of the treatment period). The study drug will be stored at room temperature in safe custody of the investigator until distribution. After the treatment period nasal sprays will be returned to the investigator.

#### Study drug formulation and dosage

*Nasonex aquosum-Nasenspray* is composed of mometasone furoate, which is delivered at 50 μg per puff, and dispersible cellulose BP 65 cps (microcrystalline cellulose, carmellose-sodium), glycerol, sodium citrate dihydrate, citric acid monohydrate, polysorbate 80, benzalkonium chloride, and phenylethyl alcohol in aqueous solution.

Patients will be instructed to administer two puffs of the nasal spray in each nostril once daily (200 μg mometasone furoate per day and placebo, respectively).

#### Compliance

Compliance of the patients will be estimated by weighing the spray bottles at the time of return.

#### Safety precautions

INCS are usually well tolerated and side effects generally are mild and reversible. Patients will be asked for possible side effects at each study visit and in the meantime telephone contact to the study personnel will be possible at any time. The drug will be discontinued if intolerance occurs and examination and treatment will be provided by an otolaryngologist if necessary.

### Concomitant medication

#### General

All additional medication being taken by the subjects on entry to the study or at any time during the study are regarded as concomitant medication and will be documented in the CRF. Concomitant medications should be kept to a minimum during the study but, if considered necessary, may be given if not interfering with the study protocol.

#### Rescue medication

Patients will be provided with one tablet of desloratadine 5 mg at the investigator’s discretion if experiencing unacceptable rhinitis symptoms at NPT.

Although anaphylactic events are very unlikely in this study but always have to be considered possible, a complete emergency kit will be available immediately in case of anaphylactic reactions at SPT or NPT.

#### Prohibited concomitant medication

Subjects must remain off medications interfering with study procedures (SPT, NPT, treatment of anaphylactic reactions) for the duration of the study. The following concomitant treatments are not permitted during this study:

- systemic or topical corticosteroids
- other immunosuppressant drugs
- antihistamines (except desloratadine 5 mg at NPTs at the investigator’s discretion) or disodium cromoglycate
- systemic or intranasal adrenergic drugs
- psychopharmacological drugs
- ACE-inhibitors or beta-blockers

If treatment with one of these drugs is considered necessary during the course of the study, subjects will be withdrawn from the study.

# Data handling procedure

Case record forms (CRFs) will be completed for each participant. Trained personnel will check the entries and any errors or inconsistencies will be corrected immediately.

# Adverse events

An adverse event is any event during a clinical study, including intercurrent illness or accident, which impairs the well-being of the subject; it may also take the form of an abnormal laboratory value. The term adverse event does not imply a causal relationship with the study treatment.

All subjects experiencing adverse events – whether considered associated with the use of the study treatment or not – will be monitored until symptoms subside and any abnormal laboratory value has returned to baseline, or until there is a satisfactory explanation for the changes observed, or until death, in which case a full pathologists report will be supplied. All findings must be reported on an “Adverse event” page in the case record form.

All adverse events will be reported on and documented as described below.

Adverse events are divided into the categories “serious” and “non-serious”. This determines the procedure which must be used to report/document the adverse event (see below).

## Definition of serious and non-serious adverse events

A serious adverse event is:

- any event that is fatal or life-threatening
- any event that is permanently disabling
- any event that requires or prolongs hospitalization
- any event that involves cancer, congenital anomaly, or occurs as a result of overdose (application of more than the stipulated dose)

Adverse events which do not fall into these categories are defined as non-serious.

## Assessment of severity of AE

Regardless of the classification of an adverse event as serious or non-serious (see above), its severity must be assessed as mild, moderate or severe, according to medical criteria alone:

mild = does not interfere with routine activities, acceptable

moderate = interferes with routine activities

severe = impossible to perform routine activities, considered as unacceptable by the physician, requires treatment, requires discontinuation of study, or has residual effect.

It should be noted that a severe adverse event needs not to be serious in nature and that a serious adverse event needs not, by definition, to be severe. Regardless of severity, all serious adverse events must be reported on as below.

## Reporting/documentation of adverse events

Adverse events are collected by spontaneous reporting.

### Reporting/documentation of serious adverse events

All serious adverse events which occur during this study, whether considered to be associated with the study medication or not, must be documented on an “Adverse event” page in the case record form.

A follow-up report including all new information obtained on the serious event must be prepared and will be collected.

The investigator will submit on request copies of all these reports to the ethics committee. Where necessary, investigators will inform the authorities.

### Reporting/documentation of non-serious adverse events

These are to be documented on an “Adverse event” page in the case record form.

# Ethical and legal aspects

The study will be carried out in keeping with local legal requirements and GCP.

## Declaration of Helsinki

The study will be performed in accordance with the guidelines of the Declaration of Helsinki (1964), including current revisions. The use of recombinant allergens for *in vitro* and *in vivo* diagnosis follows a position paper summarizing a consensus made during a WHO Meeting held in Geneva 1993 concerning this topic (Bousquet & Valenta 1994).

## Informed consent of subject

Before being admitted to the clinical study, the subject must have consented to participate after the nature, scope and possible consequences of the clinical study have been explained in a form understandable to him/her. The subject must give consent in writing. The signature of the investigator will confirm the subject’s consent. The patient may withdraw the content, even without giving comments, at any time and without negative consequences for his further medical care.

## Acknowledgement / approval of the study

Before the start of the study, the study protocol will be submitted to the Ethics committee of the Austrian Working Group for Clinical Pharmacology (Österreichische Arbeitsgemeinschaft für Klinische Pharmakologie), Kinderspitalgasse 10/15, A-1090 Vienna.

## Insurance

All subjects participating in clinical studies are insured through Generali Versicherung AG, Landskrongasse 1-3, 1011 Wien, Tel. ++43-1-534 01-4456, (insurance number: filed subsequently) in accordance with §38 of the Austrian Medicines Act (AMG).

## Confidentiality

All subjects’ names will be kept secret in the investigators files. Subjects will be identified throughout documentation and evaluation by the number allotted to them during the study. The subjects will be told that all study findings will be stored and handled in strictest confidence.

# Documentation and use of study findings

## Documentation of study findings

All results collected during the study will be entered on the CRFs. All entries on the case record forms will be made legibly in black ink. If corrections are made to entries in the case record form, the words or figures will be ringed and a single stroke drawn through them. The correct value will be entered beside the old entry and date and the correction will be initialled. Incorrect entries must not be covered with correcting fluid or obliterated, or made illegible in any way. The completed CRFs will be signed by the investigator. CRFs will be completed immediately after the final examination. The medical records upon which the CRF is based will be kept for at least 15 years.

## Use of study findings

The findings of this study will be published by the investigators in a scientific journal and presented at scientific meetings. The manuscript will be circulated to all co-investigators before submission.

# Protocol amendments

If any modifications become necessary or desirable, these will be documented in writing; major changes and changes involving patients manners require the approval of all investigators and the ethics committee.

# References

Arquint O, Helbling A, Crameri R, Ferreira F, Breitenbach M, Pichler WJ. Reduced in vivo allergenicity of Bet v 1d isoform, a natural component of birch pollen. J Allergy Clin Immunol 1999;104:1239-43.

Bachert C, Berdel D, Enzmann H, et al. Richtlinien für die Durchführung von nasalen Provokationstests mit Allergenen bei Erkrankungen der oberen Luftwege. Allergologie 1990;13:53-55.

Bachert C. Nasal provocation test: critical evaluation. New trend in allergy IV. Springer Verlag Berlin Heidelberg 1997.

Bachmann W. Die Funktionsdiagnostik der behinderten Nasenatmung. Einführung in die Rhinomanometrie. Springer Verlag Berlin 1982;1-154.

Bousquet J, Valenta R. *In vivo* and *in vitro* use of recombinant allergens. ACI News 1994;6/2:54-9.

Clement PA. Committee report on stadardization of rhinomanometry. Brussels Rhinology 1984;22:151-155.

Godnic-Cvar J, Susani M, Breiteneder H, Berger A, Havelec L, Waldhor T, Hirschwehr R, Valenta R, Scheiner O, Rudiger H, Kraft D, Ebner C. Recombinant Bet v 1, the major birch pollen allergen, induces hypersensitivity reactions equal to those induced by natural Bet v 1 in the airways of patients allergic to tree pollen. J Allergy Clin Immunol 1997;99:354-9.

Hauswirth AW, Natter S, Ghannadan M, Majlesi Y, Schernthaner GH, Sperr WR, Buhring HJ, Valenta R, Valent P. Recombinant allergens promote expression of CD 203c on basophils in sensitized individuals. J Allergy Clin Immunol. 2002;110:102-9.

Heiss S, Mahler V, Steiner R, et al. Component-resolved diagnosis (CRD) of type I allergy with recombinant grass and tree pollen allergens by skin testing. J Invest Dermatol 1999;113:830.

Henderson LL, Larson JB, Gleich GJ. Effect of corticosteroids on seasonal increases in IgE antibody. J Allergy Clin Immunol 1973;52:352-7.

Huggins KG, Brostoff J. Local production of specific IgE antibodies in allergic-rhinitis patients with negative skin tests. Lancet 1975;2:148-50.

Members of the workshop. ARIA in the pharmacy: management of allergic rhinitis symptoms in the pharmacy. Allergy 2004;59:373-387.

Mygind N, Nielsen LP, Hoffmann HJ, Shukla A, Blumberga G, Dahl R, Jacobi H. Mode of action of intranasal corticosteroids. J Allergy Clin Immunol. 2001 Jul;108(1 Suppl):S16-25. Review.

Naclerio RM, Adkinson NF Jr., Creticos PS, et al. Intranasal steroids inhibit seasonal increases in ragweed-specific immunoglobulin E antibodies. J Allergy Clin Immunol 1993;92:717-721.

Naclerio RM, Adkinson NF Jr., Moylan B et al. Nasal provocation with allergen induces a secondary serum IgE antibody response. J Allergy Clin Immunol 1997;100:505-510.

Niederberger V, Ring J, Rakoski J et al. Antigens drive memory IgE-responses in human allergy via the nasal mucosa. (submitted).

Niederberger V, Stubner P, Spitzauer S, Kraft D, Valenta R, Ehrenberger K, Horak F. Skin test results but not serology reflect immediate type respiratory sensitivity: a study performed with recombinant allergen molecules. J Invest Dermatol 2001;117:848-51.

Niederberger V, Horak F, Vrtala S, Spitzauer S, Krauth MT, Valent P, Reisinger J, Pelzmann M, Hayek B, Kronqvist M, Gafvelin G, Gronlund H, Purohit A, Suck R, Fiebig H, Cromwell O, Pauli G, van Hage-Hamsten M, Valenta R. Vaccination with genetically engineered allergens prevents progression of allergic disease. Proc Natl Acad Sci U S A. 2004;101 Suppl 2:14677-82.

Onrust SV, Lamb HM. Mometasone furoate. A review of its intranasal use in allergic rhinitis. Drugs 1998;56:725-45. Review.

Passalacqua G, Albano M, Canonica GW, Bachert C, Van Cauwenberge P, Davies RJ, Durham SR, Kontou-Fili K, Horak F, Malling HJ. Inhaled and nasal corticosteroids: safety aspects. Allergy. 2000 Jan;55(1):16-33.

Pullerits T, Praks L, Sjöstrand M, et al. An intranasal glucocorticoid inhibits the increase of specific IgE initiated during birch pollen season. J Allergy Clin Immunol 1997;100:601-605.

Riechelmann H, Bachert C, Goldschmidt O, Hauswald B, Klimek L, Schlenter WW, Tasman AJ, Wagenmann M. Durchführung des nasalen Provokationstest bei Erkrankungen der oberen Atemwege. Positionspapier der DGAKI. Allergo J 2002;11:29-36.

Sadan N, Rhyne MB, Mellits ED, Goldstein EO, Levy DA, Lichtenstein LM. Immunotherapy of pollinosis in children: investigation of the immunologic basis of clinical improvement. N Engl J Med 1969;280:623-7.

Szefler SJ. Pharmacokinetics of intranasal corticosteroids. J Allergy Clin Immunol 2001; 108(1):S26-S31.

Valenta R, Lidholm J, Niederberger V, et al. The recombinant allergen-based concept of component resolved diagnostics and immunotherapy (CRD and CRIT). Clin Exp Allergy 1999;29:896-904.

Valenta R, Sperr WR, Ferreira F, Valent P, Sillaber C, Tejkl M, Duchene M,

Ebner C, Lechner K, Kraft D, et al. Induction of specific histamine release from basophils with purified natural and recombinant birch pollen allergens. J Allergy Clin Immunol. 1993;91:88-97.

Van Cauwenberge P, Bachert C, Passalacqua G et al. Consensus statement on the treatment of allergic rhinitis. Allergy 2000;55:116-134.

Van Hage-Hamsten M, Johansson E, Roquet A, Peterson C, Andersson M, Greiff L, Vrtala S, Valenta R, Gronneberg R. Nasal challenges with recombinant derivatives of the major birch pollen allergen Bet v 1 induce fewer symptoms and lower mediator release than rBet v 1 wild-type in patients with allergic rhinitis. Clin Exp Allergy. 2002 Oct;32(10):1448-53.

Van Hage-Hamsten M, Pauli G. Provocation testing with recombinant allergens. Methods 2004;32:281-91. Review.

Vrtala S, Hirtenlehner K, Vangelista L, Pastore A, Eichler HG, Sperr WR, Valent P, Ebner C, Kraft D, Valenta R. Conversion of the major birch pollen allergen, Bet v 1, into two nonanaphylactic T cell epitope-containing fragments: candidates for a novel form of specific immunotherapy. J Clin Invest 1997;99:1673-81.

Welsh PW, Stricker WE, Chu C, et al. Efficacy of beclomethasone nasal solution, flunisolide, and cromolyn in relieving symptoms of ragweed allergy. Mayo Clin Proc 1987;62:125-134.

Wüthrich B, Schindler C, Leuenberger P, Ackermann-Liebrich U. Prevalence of atopy and pollinosis in the adult population of Switherland (SAPALDIA study). Swiss study on air pollution and lung diseases in adults. Int Arch Allergy Immunol 1995;106:149-156.

Yunginger JW, Gleich GJ. Seasonal changes in IgE antibodies and their relationship to IgG antibodies during immunotherapy for ragweed hay fever. J Clin Invest 1973;52:1268-75.

# Appendices

Appendix 1 Study flow chart

Appendix 2 Posting

**Study flow chart**

|  | Screening | Inclusion | NPT 1 | NPT 2 | 5 | 6 | 7 | 8 | 9 |
| --- | --- | --- | --- | --- | --- | --- | --- | --- | --- |
| *Time (month/ after start of study medication)* | *October / November.* | *November* | *December / d15** | *d16** | *d23** | *d30** | *d44** | *d58** | *d72** |
| Informed consent | **x** |  |  |  |  |  |  |  |  |
| Eligibility | **x** | **x** |  |  |  |  |  |  |  |
| SPT | **x** |  | **x** |  |  |  |  | **x** |  |
| Blood sampling | **x**  40 ml |  | **x**  60 ml |  | **x**  40 ml | **x**  40 ml | **x**  40 ml | **x**  60 ml | **x**  40 ml |
| Anterior rhinoscopy | **x** |  |  |  |  |  |  |  |  |
| FEV1 | **x** |  |  |  |  |  |  |  |  |
| INCS (hand-out/hand-in) |  | **x** | **x** |  |  | **x** |  |  |  |
| NPT |  |  | **x** | **x** |  |  |  |  |  |
| Check AE |  |  | **x** | **x** | **x** | **x** | **x** | **x** | **x** |
| Study completion |  |  |  |  |  |  |  |  | **x** |

**d0 = start of study medication (nasal sprays)*

**Probanden für klinische Studie gesucht**

*Titel der Studie:*

***Immunantwort von Heuschnupfenpatienten nach nasaler Provokation mit Allergenen unter Behandlung mit einem kortisonhältigen Nasenspray***

**Vorraussetzungen:**

- Alter: 18 – 50 Jahre
- saisonale, allergische Rhinitis („Heuschnupfen“) durch Gräser- und/oder Birkenpollen

**Aufwand und Entschädigung:**

- 9 Studientage (verteilt über einen Zeitraum von 3 Monaten zwischen November 2005 und Februar 2006), max. 2 Stunden/Studientag
- Aufwandsentschädigung: 200 Euro

**Für nähere Information wenden Sie sich bitte an:**

*cornelia.egger@meduniwien.ac.at*

Dr. Cornelia Egger, Institut für Pathophysiologie, Allgemeines Krankenhaus Wien

Währinger Gürtel 18-20, A-1090 Wien
