# Supplementary material for: Effects of Nasal Corticosteroids on Boosts of Systemic Allergen-Specific IgE Production Induced by Nasal Allergen Exposure
Source: PLoS One. 2015 Feb 23;10(2):e0114991. doi: 10.1371/journal.pone.0114991 (PMC4338223; doi:10.1371/journal.pone.0114991)
Supplement: S1 Study Protocol Amendment — (DOC) [file pone.0114991.s003.doc]

AMENDMENT 1

**to the protocol 03/05** (EudraCT number 2005-004274-24):

*„The effect of intranasal corticosteroids on the immune response following nasal allergen challenge in patients suffering from seasonal allergic rhinitis”*

Study drug – packaging, labelling, randomisation, and storage (6.2.1 of the protocol)

**Rationale**

The target of the study is the immune response on allergen challenge in allergic patients treated with (any) intranasal steroid vs. placebo. Initially we intended to use mometasone furoate as steroid. However, due to difficulties with the preparation of *Nasonex aquosum-Nasenspray* (mometasone propionate) and placebo blinded treatment packages early enough for conducting the study ahead of the start of the coming pollen period, *Nasonex aquosum-Nasenspray* (mometasone furoate) will be replaced by *Flixonase aquosum-Nasenspray* (fluticasone propionate), another well known nasal steroid. Fluticasone propionate and mometasone furoate applied intranasally represent equivalent drugs in terms of their safety and efficacy in the treatment of allergic rhinitis (1, 2, 3) in daily clinical practice. The study is not a company sponsored study (“Klinische Prüfung eines Arzneimittels”) but basic biomedical research on humans, according to the initial EC study application form. Therefore the target of the study not any compound stands in the focus.

Former version:

### Study drug – packaging, labelling, randomization, and storage

*Nasonex aquosum-Nasenspray* and placebo will be supplied by AESCA Ges.m.b.H., Traiskirchen, in an encoded manner. Labels will display numbers 1-60 and be randomized in blocks of 4. Sprays will be dispensed in ascending order from numbers 1-30 for mainly grass pollen allergic patients and numbers 31 to 60 for mainly birch pollen allergic patients. To prevent shortness of the drug, nasal sprays will be available twofold for each patient. Patients will hand-in the first package and be provided with the second package at NPT 1 (halftime of the treatment period). The study drug will be stored at room temperature in safe custody of the investigator until distribution. After the treatment period nasal sprays will be returned to the investigator.

#### Study drug formulation and dosage

*Nasonex aquosum-Nasenspray* is composed of mometasone furoate, which is delivered at 50 μg per puff, and dispersible cellulose BP 65 cps (microcrystalline cellulose, carmellose-sodium), glycerol, sodium citrate dihydrate, citric acid monohydrate, polysorbate 80, benzalkonium chloride, and phenylethyl alcohol in aqueous solution.

Patients will be instructed to administer two puffs of the nasal spray in each nostril once daily (200 μg mometasone furoate per day and placebo, respectively).

New version:

### Study drug – packaging, labelling, randomization, and storage

*Flixonase aquosum-Nasenspray* and placebo will be supplied by GlaxoSmithKline Pharma GmbH, Vienna, in an encoded manner. Labels will display numbers 1-60 and be randomized in blocks of 4. Sprays will be dispensed in ascending order from numbers 1-30 for mainly grass pollen allergic patients and numbers 31 to 60 for mainly birch pollen allergic patients. To prevent shortness of the drug, nasal sprays will be available twofold for each patient. Patients will hand-in the first package and be provided with the second package at NPT 1 (halftime of the treatment period). The study drug will be stored at room temperature in safe custody of the investigator until distribution. After the treatment period nasal sprays will be returned to the investigator.

#### Study drug formulation and dosage

*Flixonase aquosum-Nasenspray* is composed of fluticasone propionate, which is delivered at 50 μg per puff, and microcrystalline cellulose/carboxymethylcellulose (Avicel RC 591), dextrose, phenylethyl alcohol, diluted hydrochloric acid, polysorbate 80, and benzalkonium chloride in aqueous solution.

Patients will be instructed to administer two puffs of the nasal spray in each nostril once daily (200 μg fluticasone propionate per day and placebo, respectively).

References:

1. Corren J. Intranasal corticosteroids for allergic rhinitis: how do different agents compare? J Allergy Clin Immunol. 1999;104(4 Pt 1):S144-9.

2. Crim C, Pierre LN, Daley-Yates PT. A review of the pharmacology and pharmacokinetics of inhaled fluticasone propionate and mometasone furoate. Clin Ther. 2001;23(9):1339-54.

3. Passalacqua G, Albano M, Canonica GW, Bachert C, Van Cauwenberge P, Davies RJ, Durham SR, Kontou-Fili K, Horak F, Malling HJ. Inhaled and nasal corticosteroids: safety aspects. Allergy. 2000 Jan;55(1):16-33. Review.
