# Supplementary material for: Effects of Nasal Corticosteroids on Boosts of Systemic Allergen-Specific IgE Production Induced by Nasal Allergen Exposure
Source: PLoS One. 2015 Feb 23;10(2):e0114991. doi: 10.1371/journal.pone.0114991 (PMC4338223; doi:10.1371/journal.pone.0114991)
Supplement: S2 Study Protocol Amendment — (DOC) [file pone.0114991.s004.doc]

AMENDMENT 2

**to the protocol 03/05** (EudraCT number 2005-004274-24):

*„The effect of intranasal corticosteroids on the immune response following nasal allergen challenge in patients suffering from seasonal allergic rhinitis”*

Inclusion criteria (4.3.1 of the protocol)

**Rationale**

The primary objective of the study is to evaluate the effect of mometasone furoate *vs.* placebo applied as nasal spray on the increase of allergen specific serum IgE levels following nasal exposure to recombinant allergens. As a secondary objective we intended also to assess the allergen-specifity of the increased serum IgE levels after allergen exposure by including only double-sensitized (sensitization to rPhl p 5 *and* rBet v 1) subjects in the study. However, due to difficulties with the recruitment of enough eligible subjects according to the sample size considerations and in order not to compromise the primary endpoint of the study, subjects sensitized to one allergen only (rPhl p 5 *or* rBet v1) will be also included in the study and specific IgE levels will be accepted from 0.7 kUA/l (RAST class 2) as demonstrated by UniCAP.

Former version:

### Inclusion criteria

- sensitization to rPhl p 5 and rBet v 1 as demonstrated by UniCAP within a range from 3.0-80 kUA/l and a positive (wheal diameter >= 3 mm larger than the negative control and half of the histamine control) SPT with rPhl p 5 and rBet v 1 at 20 µg/ml

New version:

### Inclusion criteria

- sensitization to rPhl p 5 and/or rBet v 1 as demonstrated by UniCAP within a range from 0.7-80 kUA/l and a positive (wheal diameter >= 3 mm larger than the negative control and half of the histamine control) SPT with rPhl p 5 and/or rBet v 1 at 20 µg/ml
