# Supplementary material for: Effects of Nasal Corticosteroids on Boosts of Systemic Allergen-Specific IgE Production Induced by Nasal Allergen Exposure
Source: PLoS One. 2015 Feb 23;10(2):e0114991. doi: 10.1371/journal.pone.0114991 (PMC4338223; doi:10.1371/journal.pone.0114991)
Supplement: S1 Table — (PDF) [file pone.0114991.s005.pdf]

		IgE to allergen used for nasal provocation (kUA/L)						IgE to control allergen (kUA/L)					
time point:		1	2	3	4	5	6	1	2	3	4	5	6
Placebo nasal spray	nasal provocation with Phl p 5	5.51	7.15	7.37	9.98	6.90	8.16	5.50	6.49	6.21	7.27	4.83	6.05
		6.97	9.13	10.2	10.6	10.6	9.90	5.98	8.11	7.67	7.00	6.89	6.81
		7.30	8.34	11.8	12.6	11.5	11.6	26.9	31.0	45.4	39.5	39.0	40.6
		11.5	10.6	12.4	16.1	15.9	13.9	4.96	5.44	6.08	6.41	6.53	5.18
		1.18	1.06	1.61	1.90	1.76	1.85	0.68	0.62	0.79	0.59	0.59	0.64
		0.57	0.55	0.58	0.54	0.59	0.50	0.69	0.64	0.67	0.64	0.56	0.61
		38.9	37.8	58.9	84.1	94.3	72.7	18.1	17.2	17.9	19.5	18.3	14.2
		0.77	0.80	1.05	1.19	1.22	0.99	59.6	58.5	56.3	52.3	60.5	50.2
		6.95	9.27	9.05	9.64	8.04	8.38	2.96	4.05	3.25	3.09	2.75	2.42
		15.3	13.1	45.2	50.6	54.5	41.2	10.9	7.57	9.54	8.90	11.1	8.08
		37.4	28.0	29.6	31.8	31.2	29.4	21.7	16.5	15.4	15.9	15.5	14.8
		16.0	13.7	14.3	22.9	21.0	17.0	7.51	6.50	5.64	6.57	6.10	4.78
Placebo nasal spray	nasal provocation with Bet v 1	36.7	33.4	42.4	54.9	54.1	51.5	1.60	1.39	1.33	1.25	1.16	1.05
		5.38	4.59	5.14	5.93	4.96	5.19	1.34	1.06	1.11	1.26	1.05	1.11
		13.4	14.4	13.5	15.4	13.3	19.8	19.0	18.3	17.8	17.8	15.7	24.3
		44.7	36.8	53.6	50.4	44.0	42.0	12.6	10.4	10.9	9.49	8.52	9.42
		1.84	1.55	3.87	2.25	2.18	1.61	5.31	3.90	6.09	4.38	4.23	3.15
		67.5	48.7	86.4	100	100	100	40.0	28.2	28.0	31.0	27.0	28.9
		10.5	n.d.	15.7	18.9	17.9	17.2	1.94	n.d.	2.42	2.29	2.00	2.05
		0.98	0.97	0.96	0.81	0.88	0.98	15.2	16.5	14.6	12.5	12.5	14.4
		29.8	25.1	43.8	74.7	51.8	43.6	35.8	25.5	26.1	41.6	30.1	26.0
		14.9	12.9	15.6	18.2	22.8	13.8	1.65	1.18	1.32	1.51	1.95	1.05
Fluticasone nasal spray	nasal provocation with Phl p 5	34.0	28.6	37.0	49.1	40.5	35.4	0.70	0.58	0.63	0.66	0.56	0.47
		35.6	38.0	48.7	82.7	58.8	50.5	37.7	39.3	42.0	55.4	38.3	34.1
		10.8	12.5	14.3	22.2	20.5	21.1	21.6	27.6	25.9	30.4	27.2	28.4
		11.6	9.96	11.6	12.0	12.4	11.6	6.59	5.28	5.39	5.06	4.91	4.65
		11.2	9.30	53.3	100	77.5	76.5	5.62	4.60	5.17	7.60	5.34	5.33
		43.4	39.4	49.3	54.3	43.9	43.2	7.40	6.48	8.29	9.67	8.29	7.03
		21.1	24.8	23.7	29.8	25.8	n.d.	6.86	8.52	8.16	8.23	7.37	n.d.
		26.6	n.d.	49.9	46.1	37.7	47.1	7.96	n.d.	12.0	6.59	5.95	7.90
		10.7	9.72	12.2	15.6	15.7	15.3	7.75	7.31	7.13	6.58	6.16	6.50
		10.6	7.61	11.4	18.2	13.9	14.1	7.89	5.63	6.11	6.64	5.15	5.22
		14.4	9.85	15.4	24.0	18.5	19.0	17.1	11.5	11.8	14.2	10.5	10.9
		45.9	45.6	73.1	100	100	98.3	27.0	26.4	31.7	34.1	28.9	27.2
		2.39	2.09	3.97	5.26	4.68	4.17	0.35	0.35	0.35	0.35	0.35	0.35
Fluticasone nasal spray	nasal provocation with Bet v 1	54.1	45.0	64.1	99.0	94.2	84.8	0.86	0.62	0.59	0.74	0.64	0.68
		26.0	20.8	22.7	29.9	27.9	25.9	22.1	16.3	16.8	17.7	16.5	16.1
		5.89	5.74	5.89	5.09	4.96	5.28	100	100	96.2	85.2	79.1	82.9
		19.7	13.4	14.7	13.7	13.5	10.9	0.60	0.43	0.42	0.38	0.39	0.35
		5.15	4.77	10.7	18.2	14.7	13.2	2.07	1.49	1.57	1.86	1.49	1.35
		0.84	0.60	0.95	1.08	1.13	0.86	3.82	2.92	2.61	2.25	2.29	1.82
		1.85	3.79	7.29	8.83	7.03	7.35	3.68	5.85	6.68	7.28	5.65	6.18
		6.34	6.76	6.49	7.58	9.11	6.42	4.68	5.48	4.70	4.99	6.17	4.43
		12.7	11.5	10.1	11.5	10.9	11.0	5.17	4.87	3.96	4.28	3.79	4.27
		5.56	4.98	4.54	4.87	5.86	6.89	8.00	7.27	6.85	7.57	8.21	9.72

time points:

1 immediately before nasal provocation
2 1 week after nasal provocation
3 2 weeks after provocation
4 4 weeks after provocation
5 6 weeks after provocation
6 8 weeks after provocation
